# Cell Surface Properties of *Lactococcus lactis* Reveal Milk Protein Binding Specifically Evolved in Dairy Isolates

**DOI:** 10.3389/fmicb.2017.01691

**Published:** 2017-09-07

**Authors:** Mariya Tarazanova, Thom Huppertz, Marke Beerthuyzen, Saskia van Schalkwijk, Patrick Janssen, Michiel Wels, Jan Kok, Herwig Bachmann

**Affiliations:** ^1^NIZO Ede, Netherlands; ^2^TI Food and Nutrition Wageningen, Netherlands; ^3^Molecular Genetics, University of Groningen Groningen, Netherlands

**Keywords:** gene-trait matching, cell surface hydrophobicity, surface charge, attachment to milk proteins, emulsion stability, bacteria-protein interactions, cell wall composition, *Lactococcus lactis*

## Abstract

Surface properties of bacteria are determined by the molecular composition of the cell wall and they are important for interactions of cells with their environment. Well-known examples of bacterial interactions with surfaces are biofilm formation and the fermentation of solid materials like food and feed. *Lactococcus lactis* is broadly used for the fermentation of cheese and buttermilk and it is primarily isolated from either plant material or the dairy environment. In this study, we characterized surface hydrophobicity, charge, emulsification properties, and the attachment to milk proteins of 55 *L. lactis* strains in stationary and exponential growth phases. The attachment to milk protein was assessed through a newly developed flow cytometry-based protocol. Besides finding a high degree of biodiversity, phenotype-genotype matching allowed the identification of candidate genes involved in the modification of the cell surface. Overexpression and gene deletion analysis allowed to verify the predictions for three identified proteins that altered surface hydrophobicity and attachment of milk proteins. The data also showed that lactococci isolated from a dairy environment bind higher amounts of milk proteins when compared to plant isolates. It remains to be determined whether the alteration of surface properties also has potential to alter starter culture functionalities.

## Introduction

The bacterial surface is important for interactions of the cell with the environment, especially when it comes to surface adhesion (Bellon-Fontaine et al., 1996; Ly et al., 2006; Boks et al., 2008). Examples for such interactions are the fermentation of solid substrates like fermented foods (Sieuwerts et al., 2008), woody materials and straw (Bayer et al., 2004), bioremediation of soil (Groudev et al., 2010), the formation of biofilms (Decho, 2000; Sutherland, 2001; Prouty et al., 2002; Newman et al., 2013) or during attachment of bacterial cells to the intestinal tract (Kleerebezem and Vaughan, 2009; Bron et al., 2012). Microbial surface properties are especially important for the initial contact with and adhesion to a surface, which can occur via fimbriae, pili, flagella, or EPS (Van Houdt and Michiels, 2010). Once attached, cells can start to produce different polymeric components such as polysaccharides, glycoproteins, proteins, glycolipids, cellulose, and extracellular DNA (Van Houdt and Michiels, 2010), which can lead to biofilm formation and can further accelerate bacterial adhesion (Kumar and Anand, 1998; Decho, 2000).

The cell surface itself is characterized by properties like charge and hydrophobicity (Ly et al., 2008), which are determined by the molecular composition of the cell wall. The cell wall consists of peptidoglycan (Chapot-Chartier and Kulakauskas, 2014), polysaccharides (Ruas-Madiedo et al., 2002), proteins, teichoic, and lipoteichoic acids, lipids (Pelletier et al., 1997) and can be decorated with a sugar pellicle (Chapot-Chartier et al., 2010), pili (Telford et al., 2006; Oxaran et al., 2012; Meyrand et al., 2013; Castelain et al., 2016), and/or an S-layer (van der Mei et al., 2003). The charge of the cell surface is determined by positively and negatively charged groups on teichoic and lipoteichoic acids, polysaccharides, proteins, and pili (Delcour et al., 1999; Boonaert and Rouxhet, 2000; Chapot-Chartier et al., 2010), while its hydrophobicity is related to the presence of polysaccharides, LPS, and (glyco-) proteinaceous material (Pelletier et al., 1997; Firoozmand and Rousseau, 2016), as well as pili (Tarazanova et al., 2016). Although the bacterial surface contains positively and negatively charged molecules, the net surface charge of bacteria is mostly negative (Neu and Marshall, 1990). The surface composition is species and strain specific (Wicken et al., 1983), and can vary between different growth substrates (Wicken et al., 1983) and growth phases (Boonaert and Rouxhet, 2000; Schär-Zammaretti and Ubbink, 2003).

The interactions between cell surface and substrate can be electrostatic. For example, in sand, a strong negative charge of the cell surface causes electrostatic repulsion and thus prevents bacterial adhesion and increases cell transport through the sand matrix while cells with high hydrophobicity are retained by that matrix (Jacobs et al., 2007). Other types of interactions occurring are hydrophobic, van der Waals, and Lewis acid–base forces. An example is the biofilm formation in which Brownian motion, hydrogen bonding, and electrostatic forces play a predominant role during the initial cell attachment, while cell hydrophobic forces as well as dipole-dipole, ionic bonding become more prominent during the phase of “irreversible” attachment of the bacteria to the surface (Neu and Marshall, 1990; Kumar and Anand, 1998). In other words, during bacteria-substrate interactions, a combination of all forces is present: at initial interactions long-range forces are most important but once attachment is achieved, the short-range forces may predominate. Factors like pH, temperature and ionic strength influence the interactions and add complexity to explanations of bacteria-substrate interactions.

*Lactococcus lactis* is widely used as a starter culture in the production of cheese, sour cream, and buttermilk (Leroy and De Vuyst, 2004), where it is responsible for food preservation, flavor formation, and textural properties (Leroy and De Vuyst, 2004). It is classified into the subspecies (ssp.) *lactis* including ssp. *lactis* biovar. *diacetylactis*, ssp. *cremoris*, and ssp. *hordniae*. The molecular composition of the *L. lactis* cell wall and its interactions with food components were reviewed by Burgain et al. (2014). It was shown that within *L. lactis* the diversity in cell surface charge, hydrophobicity and the ability to stabilize emulsion is very high (Ly et al., 2006). Most *L. lactis* strains originate either from a dairy environment or from plant material, and literature suggests that strains of dairy origin have evolved from plant isolates (van Hylckama Vlieg et al., 2006). The transition from the plant to the dairy environment was analyzed by comparative genomics (Siezen and van Hylckama Vlieg, 2011) or experimental evolution (Bachmann et al., 2012) and the results consistently describe similar metabolic adaptations. The main alterations during the plant-dairy transition are the loss of genes for the utilization of carbohydrate those only occur in plant material and the improved utilization of milk proteins. However, nothing is known about possible effects of the environmental transition on surface properties.

In this study, we investigated the surface properties of 55 *L. lactis* strains of which 25 were isolated from plant material and 30 from a dairy environment. We measured the cell surface hydrophobicity (CSH) and charge as well as emulsion stabilizing properties and the attachment of the bacterial cells to milk proteins. Genotype-phenotype matching (GTM) (Siezen et al., 2008; Bayjanov et al., 2012, 2013) allowed identifying key molecules involved in *L. lactis* surface properties. An analysis based on phylogeny and strain origin revealed that dairy isolates have a much higher capacity to bind milk proteins.

## Materials and methods

### Bacterial strains and culturing conditions

*L. lactis* used in this study (Table 1) were grown as standing cultures at 30°C in M17 (Oxoid, Thermo Scientific, Hampshire, UK) broth supplemented with 1% glucose (GM17) or 1% lactose (LM17). *E. coli* E10 containing pUC19 with an erythromycin resistance gene, pUC19E, was grown in tryptone yeast extract broth (TYB) at 37°C under vigorous shaking and access of oxygen. When required, antibiotics were added to the media: erythromycin (Em) was used at 10 μg/ml; chloramphenicol (Cm)—10 μg/ml; nisin—10 ng/ml. Optical density at 600 nm (OD_600_) was measured using a single cell spectrophotometer (Ultrospec 2000, Pharmacia Biotech, Centerville, USA). Exponentially growing cells were prepared by diluting an overnight culture to an OD_600_ of 0.01 and subsequent incubation until an OD_600_ of 0.45 ± 0.04 was reached. Stationary cells were prepared similarly by growing a culture for 16–18 h.

**Table 1:** The 55 *L. lactis* strains and plasmid used in this study.

	**Strain**	**Genotype**	**Origin**	**References**
1	ATCC19435	*L. lactis* ssp. *lactis*	Milk (dairy starter)	Siezen et al., 2011; Backus et al., 2017
2	HP	*L. lactis* ssp. *cremoris*	Dairy starter	Siezen et al., 2011; Wels et al., 2017
3	P7266	*L. lactis* ssp. *lactis*	Litter on pastures	Siezen et al., 2011; Backus et al., 2017
4	NCDO895	*L. lactis* ssp. *lactis*	Dairy starter	Siezen et al., 2011; Backus et al., 2017
5	LMG8520	*L. lactis* ssp. *Hordniae*	Leaf hopper	Siezen et al., 2011; Backus et al., 2017
6	N41	*L. lactis* ssp. *cremoris*	Soil and grass	Siezen et al., 2011; Wels et al., 2017
7	M20	*L. lactis* ssp. *lactis* biovar. *diacetylactis*	Soil	Siezen et al., 2011; Backus et al., 2017
8	ML8	*L. lactis* ssp. *lactis*	Dairy starter	Siezen et al., 2011; Backus et al., 2017
9	V4	*L. lactis* ssp. *cremoris*	Raw sheep milk	Siezen et al., 2011; Wels et al., 2017
10	Li-1	*L. lactis* ssp. *lactis*	Grass	Siezen et al., 2011; Backus et al., 2017
11	UC317	*L. lactis* ssp. *lactis*	Dairy starter	Siezen et al., 2011; Backus et al., 2017
12	E34	*L. lactis* ssp. *lactis*	Silage	Siezen et al., 2011; Backus et al., 2017
13	N42	*L. lactis* ssp. *lactis*	Soil and grass	Siezen et al., 2011; Backus et al., 2017
14	DRA4	*L. lactis* ssp. *lactis* biovar. *diacetylactis*	Dairy starter	Siezen et al., 2011; Backus et al., 2017
15	AM2	*L. lactis* ssp. *cremoris*	Dairy starter	Siezen et al., 2011; Wels et al., 2017
16	P7304	*L. lactis* ssp. *lactis*	Litter on pastures	Siezen et al., 2011; Backus et al., 2017
17	LMG8526	*L. lactis* ssp. *lactis*	Chinese radish seeds	Siezen et al., 2011; Backus et al., 2017
18	LMG9446	*L. lactis* ssp. *lactis*	Frozen peas	Siezen et al., 2011; Backus et al., 2017
19	LMG9447	*L. lactis* ssp. *lactis*	Frozen peas	Siezen et al., 2011; Backus et al., 2017
20	LMG14418	*L. lactis* ssp. *lactis*	Bovine milk	Siezen et al., 2011; Backus et al., 2017
21	NIZO2244B	*L. lactis* ssp. *lactis*	Mustard and cress	Siezen et al., 2011; Backus et al., 2017
22	FG2	*L. lactis* ssp. *cremoris*	Dairy starter	Siezen et al., 2011; Wels et al., 2017
23	K231	*L. lactis* ssp. *lactis*	White kimchi	Siezen et al., 2011; Backus et al., 2017
24	KF7	*L. lactis* ssp. *lactis*	Alfalfa sprouts	Siezen et al., 2011; Backus et al., 2017
25	KF24	*L. lactis* ssp. *lactis*	Alfalfa sprouts	Siezen et al., 2011; Backus et al., 2017
26	KF146	*L. lactis* ssp. *lactis*	Alfalfa and radish sprouts	Siezen et al., 2011; Backus et al., 2017
27	KW10	*L. lactis* ssp. *cremoris*	Kaanga way	Siezen et al., 2011; Wels et al., 2017
28	K337	*L. lactis* ssp. *lactis*	White kimchi	Siezen et al., 2011; Backus et al., 2017
29	KF67	*L. lactis* ssp. *lactis*	Grapefruit juice	Siezen et al., 2011; Backus et al., 2017
30	KF134	*L. lactis* ssp. *lactis*	Alfalfa and radish sprouts	Siezen et al., 2011; Backus et al., 2017
31	KF196	*L. lactis* ssp. *lactis*	Japanese kaiwere shoots	Siezen et al., 2011; Backus et al., 2017
32	KF201	*L. lactis* ssp. *lactis*	Sliced mixed vegetables	Siezen et al., 2011; Backus et al., 2017
33	KF282	*L. lactis* ssp. *lactis*	Mustard and cress	Siezen et al., 2011; Backus et al., 2017
34	LMG6897	*L. lactis* ssp. *cremoris*	Cheese starter	Siezen et al., 2011; Wels et al., 2017
35	NCDO763	*L. lactis* ssp. *cremoris*	Dairy starter	Siezen et al., 2011; Wels et al., 2017
36	SK11	*L. lactis* ssp. *cremoris*	Dairy starter	Siezen and Renckens, 2005; Wels et al., 2017
37	MG1363	*L. lactis* ssp. *cremoris*	Cheese starter	Wegmann et al., 2007
38	KF147	*L. lactis* ssp. *lactis*	Mung bean sprouts	Siezen et al., 2010; Backus et al., 2017
39	IL1403	*L. lactis* ssp. *lactis*	Dairy starter	Bolotin et al., 2001
40	MG1299	*L. lactis* ssp. *cremoris*	Dairy starter	Wegmann et al., 2012
41	B40	*L. lactis* ssp. *cremoris*	Dairy starter	van Kranenburg et al., 1997; Wels et al., 2017
42	NCDO712	*L. lactis* ssp. *cremoris*	Dairy starter	Tarazanova et al., 2016
43	SK110	*L. lactis* ssp. *cremoris*	Dairy starter	Sijtsma et al., 1988; Wels et al., 2017
44	MG1362	*L. lactis* ssp. *cremoris*	Dairy starter	Gasson, 1983
45	MG1063	*L. lactis* ssp. *cremoris*	Dairy starter	Gasson, 1983
46	MG1261	*L. lactis* ssp. *cremoris*	Dairy starter	Gasson, 1983
47	MG1365	*L. lactis* ssp. *cremoris*	Dairy starter	Gasson, 1983
48	TIFN1	*L. lactis* ssp. *cremoris*	Dairy starter	Erkus et al., 2013
49	TIFN2	*L. lactis* ssp. *lactis* biovar. *diacetylactis*	Dairy starter	Erkus et al., 2013
50	TIFN3	*L. lactis* ssp. *cremoris*	Dairy starter	Erkus et al., 2013
51	TIFN4	*L. lactis* ssp. *lactis* biovar. *diacetylactis*	Dairy starter	Erkus et al., 2013
52	TIFN5	*L. lactis* ssp. *cremoris*	Dairy starter	Erkus et al., 2013
53	TIFN6	*L. lactis* ssp. *cremoris*	Dairy starter	Erkus et al., 2013
54	TIFN7	*L. lactis* ssp. *cremoris*	Dairy starter	Erkus et al., 2013
55	NZ9000	*L. lactis* ssp. *cremoris*	Dairy starter	Linares et al., 2010
**PLASMID USED FOR GENE OVER-EXPRESSION**				
	**Plasmid**	**Host organism**		**Reference**
	pNZ8150	*L. lactis*		Mierau and Kleerebezem, 2005

### Cell surface charge (mV)

Cells were harvested by centrifugation at 2,676 g for 3 min at room temperature and the cell pellet was washed 2x with 1 volume of 10 mM phosphate buffer (PB; pH = 6.7) and re-suspended in the same buffer to an optical density OD_600_ of 1. Approximately 2 ml of this cell suspension was filled into the ZetaSizer DST1070 cuvette, which was inserted into ZetaSizer (Nano-ZS, Malvern, Malvern, UK). The electrophoretic mobility of cells was measured at 20°C and automatically re-calculated into the values of zeta potential (mV).

### Cell surface hydrophobicity (CSH, %)

Cell surface hydrophobicity (CSH, %) was measured as described previously (Rosenberg et al., 1980) with the following modifications: 5 ml of cell suspension (OD_600_ = 1) in PB was mixed with 2 ml of either petroleum or hexane (both from Sigma-Aldrich Chemie Gmbh, Munich, Germany) in surfactant-free glass tubes with a surfactant-free stopper. Tubes were vortexed for 2 min and kept still for 15 min at room temperature to allow phase separation to occur. Subsequently, 1 mL of the aqueous phase was transferred to a spectrophotometer cuvette and optical density (OD_600_) of the cell suspension was measured at 600 nm. The surface hydrophobicity was calculated according to the following formula:

Cell Surface Hydrophobicity (CSH, %) = (A_0_−A_1_)/A_0_·100, in which A_0_ represents the initial OD_600_ of cell suspension before mixing and A_1_ is the OD_600_ of the water phase after mixing with petroleum or hexane and subsequent phase separation.

### Emulsion stability (E24, %)

Emulsion Stability (E24, %) was determined as described earlier (Khopade et al., 2012; Padmapriya, 2012). Initially samples of cells in exponential and stationary growth phases were prepared in the same way as described for CSH with slight modifications. Briefly, 5 ml of cell suspension (OD_600_ = 1) in PB was mixed with 2 ml hydrocarbon, vortexed for 2 min and left standing for 24 h. The E24 index is given as a percentage according to: E24(%) = h_1_/h_2_*100%, in which h_1_ is the height of the emulsified layer and h_2_ is the total height of the emulsified layer and liquid column, both in mm.

### Cell binding capacity to milk proteins

To 1 ml of cell suspension in either stationary or exponential growth phase prepared as described above (OD_600_ = 1) 1 μL Syto 9 was added (Green Fluorescent Nucleic Acid Stain, Life Technologies, Bleiswijk, The Netherlands) after which the suspension was incubated in the dark for 30 min at room temperature. The cells were washed twice with PB to remove free dye.

#### Preparation of proteins

Sodium caseinate and sodium para-caseinate suspensions were prepared by dissolving 10 g of protein powder in 100 ml sterile demineralized water, followed by incubation for 20 min at 30–40°C to bring the proteins into solution and adjustment of the pH to 6.7 with 0.1 M NaOH or with 0.1 M HCl. One half of the prepared sodium caseinate solution was heated to 90°C for 10 min which allows denaturation of the residual whey proteins and their interaction with caseins. For protein staining 400 μl Nile blue A (Sigma-Aldrich Chemie Gmbh) was added to 100 ml of each protein solution, mixed, and incubated for 15 min at 21°C in the dark. To remove surplus dye the protein solution was transferred to a cellulose membrane tube with a molecular weight cut-off of 14 kDa (Sigma-Aldrich D9777-100FT, 25 mm width, 60 cm in length). Membrane tubes were pre-soaked in the sterile demineralized water for 1 h at room temperature. The protein solution in the membrane tube was dialyzed against sterile PB for 24 h at 4°C in the dark. After dialysis, the protein concentration was quantified with Pierce BCA Protein Assay Kit (ThermoFisher, Bleiswijk, The Netherlands) according to the manufacturer's instructions. The Nile blue A-stained protein solution was divided over sterile Eppendorf tubes and stored at −40°C. The protein solutions were diluted to a final concentration of 1% prior to using them in the experiments.

For attachment measurements, 0.1 ml of Syto9-stained cells and 0.1 ml of Nile blue A-stained proteins were mixed with a vortex for 10–15 s and incubated for 1 min. Subsequently, this solution was analyzed in a Flow Cytometer (BD FACSaria II Cell Counter, BD BioSciences, Sparks, MD, USA). Excitation/emission wavelengths were 635/660 ± 20 nm for Nile blue A and 485/530 ± 30 nm for Syto 9, respectively. The results were analyzed using Flowing Software version 2.5.0 (http://www.flowingsoftware.com/).

#### Sorting procedure

Automatic (CST) and “Accudrop Drop Delay” calibration (BD BioSciences, USA) of the flow cytometer was performed with 70 μm nozzle and threshold for FSC and SSC of 1,500. A total of 10,000 events from the area of interest were sorted in 1 ml of sterile PB. Serial dilutions (10^−1^, 10^−2^, 10^−3^) of the sorted events were prepared in the sterile PB and 100 μl of each dilution was plated on LM17 or GM17 agar plates. The agar plates were incubated overnight at 30°C and colony counts were determined.

### Fluorescence microscopy

Cells and proteins were stained as described above; 100 μl of stained cells (OD_600_ = 1) were mixed with the same volume of 1% protein solution. Subsequently, 1–2 μl of this mixture was placed on a microscope slide, covered with a cover slip and examined at 100-fold magnification using an Olympus BX41 microscope (Olympus Corporation, Tokyo, Japan) with excitation wavelengths of 485 ± 10 and 635 ± 10 nm and emission wavelengths of 530 ± 30 and 660 ± 20 nm, respectively. Images were acquired with a charge-coupled-device camera with identical acquisition settings for all images; exposure to excitation light was for 200 ms for the Syto 9-stained cells and for 2,000 ms for the Nile Blue A-stained proteins. Image overlays were generated using ImageJ version 1.45s (https://imagej.nih.gov/ij).

### Data analysis

Gene-trait matching (GTM) was performed using PhenoLink (Bayjanov et al., 2013). Data visualization was done using R (https://cran.r-project.org/bin/windows/base/). The heatmap.plus function using Euclidian distance matrices and average hierarchical clustering and data scaling was used for the generation of heat maps.

### Gene overexpression or deletion

Genes targeted for overexpression were PCR amplified using the hot-start KOD polymerase (Novagen, Madison, USA) according to the protocol of the manufacturer with primers listed in Table S1. Amplicons were purified using MSB® Spin PCRapase (Invitek, Gmbh, Berlin, Germany), digested with the restriction enzymes ScaI and XbaI (Fermentas GmbH, St. Leonn-Rot, Germany) and ligated into plasmid pNZ8150 (Table 1) digested with the same enzymes using T4 DNA ligase (Invitrogen, Breda, the Netherlands). DNA purification was carried out according to the protocols supplied by the manufacturers with Wizard®SV gel and PCR Clean-Up system (Promega, Leiden, The Netherlands). Ligations were carried out at 16°C for 16–18 h and the ligated product, which was precipitated with 3 M sodium acetate (pH 5.5) and 70% ethanol, was used to transform electrocompetent cells of *L. lactis* NZ9000 (Holo and Nes, 1989). After electroporation, cells were plated on GM17 agar plates containing 10 μg/ml chloramphenicol and incubated at 30°C for 3 days. Single colonies were isolated and insert DNA in the plasmid was confirmed using colony PCR with the appropriate primers (Table S1A).

Double crossover knock-outs of 4 genes were made in *L. lactis* MG1363 using pUC19 harboring an erythromycin (Em) resistance gene, pUC19E. Upstream and downstream flanking regions (left flank, LF; right flank, RF) of the target genes were amplified with the primer pairs described in Table S1B. Typically the left amplified flanking regions contained a sequence overlapping with the right flanking region (termed +vlag in the primer name in Table S1B) which allowed to perform a splicing by overlap extension (SOE) PCR (Horton et al., 2013) to generate amplicons which were digested and subsequently ligated into similarly digested pUC19E. The ligation mixture was used to transform *L. lactis* MG1363 as described previously (Holo and Nes, 1989). Strains that were the result of a single crossover event were selected after plating and incubating the transformation mixture for 2 days at 30°C on GM17 agar plates supplemented with erythromycin. These single cross-over strains were grown for at least 100 generations in GM17 broth without Em to obtain strains that were the results of a double crossover event and that became Em-sensitive. The presence of the correct, clean gene deletions was confirmed by PCR using specific primers (Table S1B).

Protein overexpression was verified with Sodium Dodecyl Sulfate—(10%) Polyacrylamide Gel Electrophoresis (SDS-PAGE) according to NuPAGE technical guide (Invitrogen, Carlsbad, CA) in cell extracts and supernatant fractions of exponentially growing cells (OD_600_ = 0.42–0.45) grown for 5h after addition of 10 ng/ml nisin.

Cell extracts were obtained by collecting the cell pellet from 10 ml culture (2,927 g for 5 min) and protein in supernatants was precipitated using trichloroacetic acid (TCA). The cell pellet was re-suspended in sterile demineralized water to a final OD_600_ of 5 and transferred to microfuge tubes containing 1 g of zirconium beads and cooled on ice for 5 min. This was followed by 3 × 30 s of bead beating with a FastPrep FP100 bead-beater (Qbiogene, Cedex, France) and 1 min off cooling on ice in between the three cycles. After this treatment the tubes were left on ice for 5 min to allow the beads to sink and the supernatant was transferred to sterile Eppendorf tubes and kept at −20°C.

TCA precipitation was conducted by adding 2.5 ml 100% trichloracetic acid to 10 ml of culture supernatant, followed by vigorous mixing and incubation for 30 min on ice. Subsequently the tubes were centrifuged at 2,927 g at 4°C for 30 min, the supernatant was discarded carefully and the pellet was dissolved in 0.5 ml cold acetone. This was followed by centrifugation at the 2,081 g at 4°C for 15 min, acetone was discarded; pellet was dried on air and r-suspended in sterile reverse osmosis water.

## Results

### Bacteria-protein interactions

**Figure 1:**
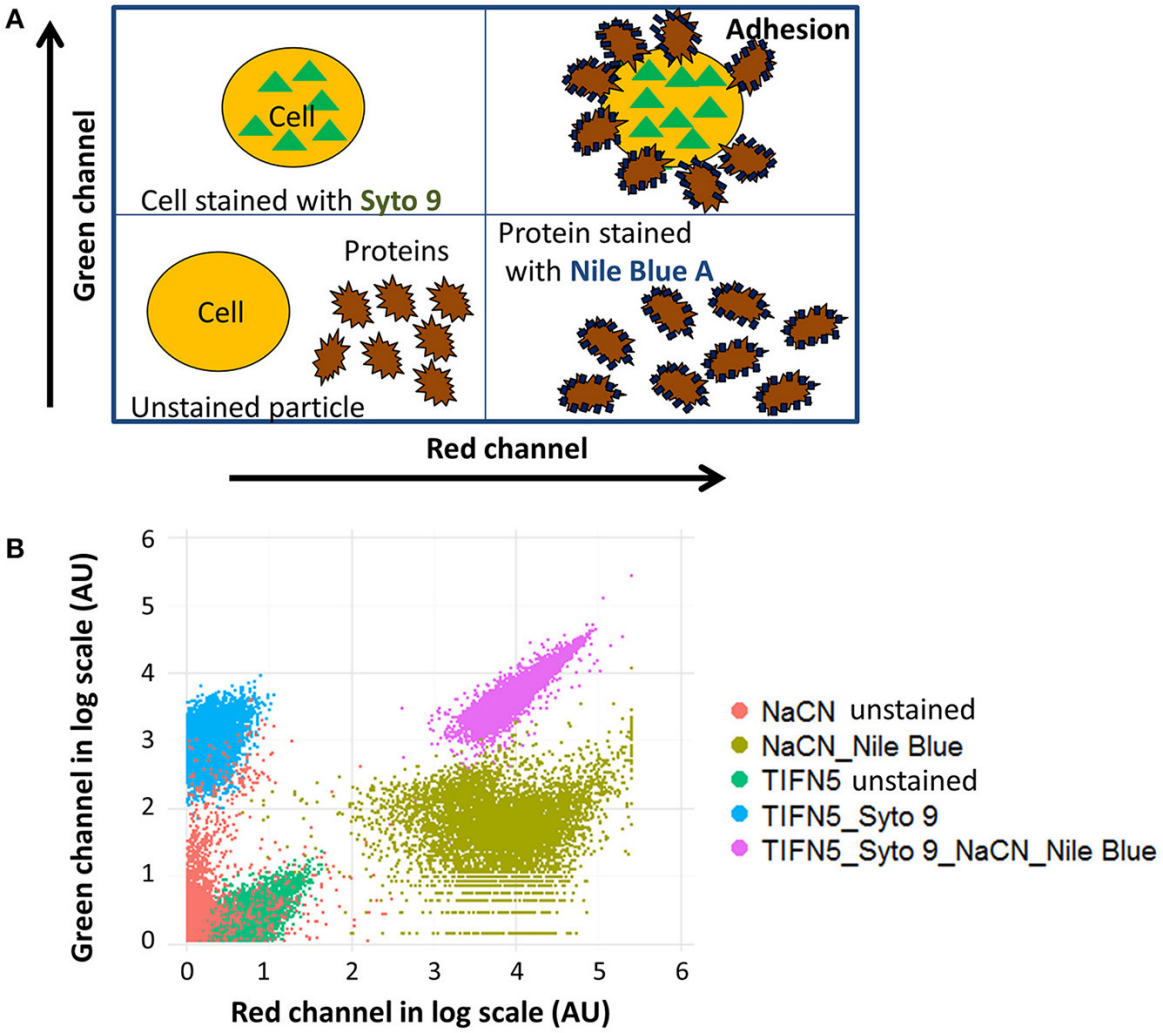
Measuring protein attachment to bacteria using flow cytometry. **(A)** Schematic view of bacteria-protein interaction and their expected appearance in a flow cytometer measurement. Unstained proteins and cells are expected in the lower left quadrant. The lower right quadrant shows proteins stained with a red fluorescent dye while the upper the left quadrant shows cells stained with a green fluorescent dye. Bacteria covered with surface-bound protein should appear in the upper right quadrant while two separate clouds should be seen if the proteins do not attach to the cell surface. **(B)** Example of attachment of sodium caseinate (NaCN) to *L. lactis* TIFN5. Values on the both axes are log-transformed. Unstained proteins (red) and cells (dark green) are located in the bottom left quadrant; stained cells are shown in blue, stained protein are colored light green, and events representing proteins-attached-to-cells are located in the upper right corner (purple).

**Figure 2:**
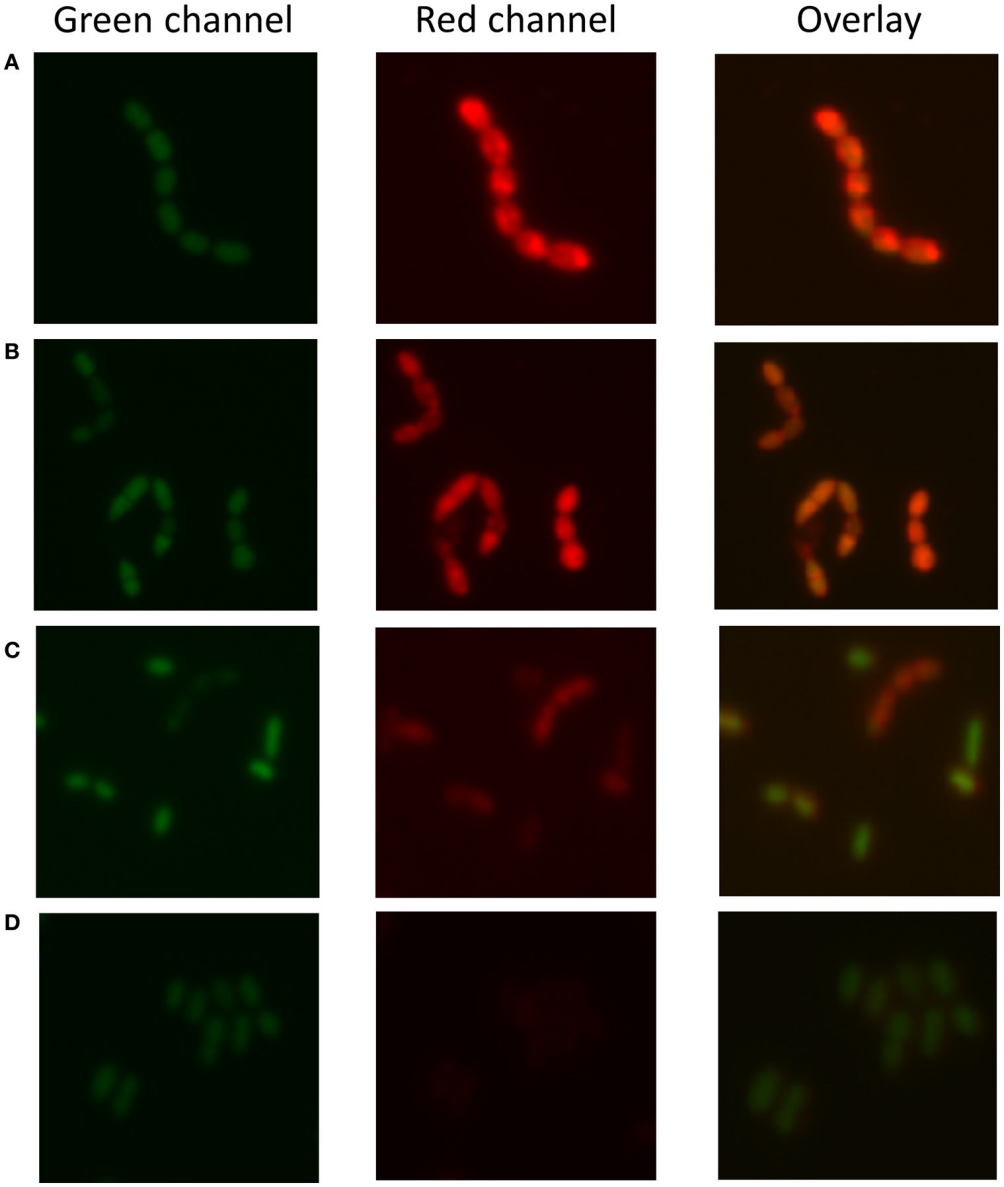
Fluorescence microscopy to determine protein (sodium caseinate) attachment to *L. lactis*. Strains are sorted from **(A–D)** by decreasing attachment strength as determined by the flow cytometry-based method. **(A)** *L. lactis* HP, **(B)** *L. lactis* SK11, **(C)** *L. lactis* KW10, **(D)** *L. lactis* P7266. The green channel represents the DNA stain the red channel the stained protein bound to the cell surface and the overlay combines both signals. The results indicate decreased attachment of sodium caseinate to the strains from **(A–D)**, respectively, which is consistent with the findings of the flow cytometric approach.

As 30 out of 55 strains used in this study were isolated from a dairy environment, we decided to examine the affinity of *L. lactis* to different milk proteins. For this purpose, we developed a protocol that allowed quantifying the attachment of proteins to microbial cells. Bacterial cells were stained with the fluorescent DNA stain Syto 9, while proteins were colored with the fluorescent dye Nile Blue A. The emission spectra of these two dyes show little overlap and the individually stained particles could thus be distinguished by flow cytometry (Figure 1A). Cells give a high fluorescent emission signal at 530 nm while the proteins emit the highest signal at 680 nm. When Nile Blue A-stained protein binds to a Syto 9-labeled bacterial cell, the resulting particle should show high fluorescence at both wavelengths while this should not be the case when the two do not interact. This basic concept was tested using *L. lactis* TIFN5 (Figure 1B). We performed two additional experiments to validate the methodology with independent techniques. In the first experiment, Syto-9 labeled cells were mixed with stained proteins. The strain used was predicted to give limited to no binding to the protein based on flow cytometry results. Using the flow cytometer we sorted 10,000 events from the population identified as protein based on the described method. The plating of these proteins showed that ~5% of the sorted events led to the formation of a colony. On the other hand, sorting of events from the population identified as cells resulted in ~95% of the cases leading to growth of a colony. These results were consistent with what was expected with an acceptable error margin of ~5%. In the second validation experiment, cells that were identified by flow cytometry as either binding or not binding to milk proteins were incubated with the protein of interest and subsequently visualized by light microscopy (Figure 2). Clear differences in Nile Blue A fluorescence intensity indicate different levels of protein binding to the cells. The level of protein binding to a cell, as detected by fluorescence microscopy at 680 nm (Figure 2), is in agreement with the protein binding levels observed with flow cytometry, which decreases from strain HP to SK11, KW10 and P7266 respectively (Figure S2, Table S2).

Sodium caseinate and sodium para-caseinate were selected as the proteins to be studied, as they represent the major milk proteins, i.e., the caseins. In sodium para-caseinate, the C-terminal caseinomacropeptide (CMP) of κ-casein is removed by enzymatic hydrolysis, as in cheese-making. Sodium caseinate was used in two forms: either or not pre-treated for 10 min at 90°C. The heat treatment was performed to account for possible heat-induced changes in the proteins, which is a common processing step when manufacturing fermented milk products (Hashizume and Sato, 1988; Lucey and Singh, 1997).

**Figure 3:**
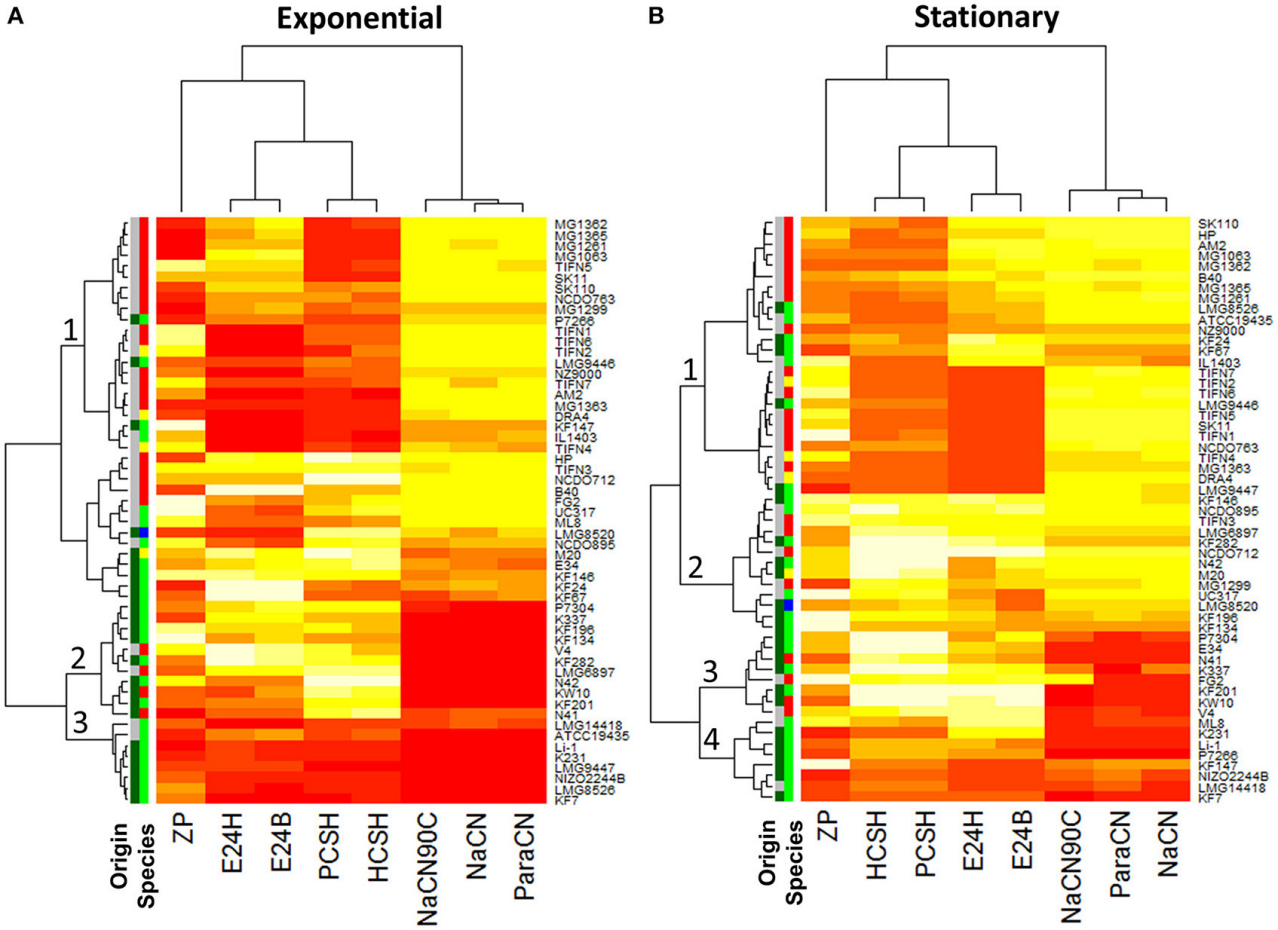
Heat map of surface properties of 55 *Lactococcus lactis* strains: cell charge (ZP), hydrophobicity measured with petroleum (PCSH) or hexane (HSCH), emulsion stability measured after 24 h (E24B—measured with Petroleum or E24H—measured with Hexane), attachment to milk proteins: para-caseinate (ParaCN), sodium caseinate (NaCN), and sodium caseinate heated for 10 min at 90°C (NaCN90C). **(A)** Comprises results using the cells from the exponential growth phase, while for **(B)** cells from the stationary growth phase were taken. Low values are represented by a darker/red color while higher values are represented by a lighter/yellow color of the heat map segment (*n* = 3). For the charge (ZP) a darker color represents more negatively charged cells. The Origin/Species columns, respectively, indicate plant (green) or dairy (gray) origin and the species *lactis* (green), *cremoris* (red), *hordniae* (blue), or *lactis* biovar *diacetylactis* (yellow).

Using this approach, 55 strains of *L. lactis* were screened for their ability to bind the three milk proteins. The strains bound all proteins to some extent, but clear differences were observed (Table S2, Figure 3, Figures S1–S3). Significant differences in growth phase-dependent protein binding were seen for 12 of the 55 strains (Figure S4). While the ability of *L. lactis* to bind proteins is strain-specific the results also show that the capacity of binding milk proteins appeared to be larger than 61% with the average between 82 and 97% for strains of dairy origin. These mostly belong to the *L. lactis* ssp. *cremoris* and ssp. *lactis* biovar. *diacetylactis*. The 10 out of 23 *L. lactis* strains of plant origin, which belong to the ssp. *lactis* (indicated as dark and light green in the “origin” and “species” column in Figure 3, respectively), showed either poor (<50%) or no protein binding for both growth phases (Figure 3, Figures S1–S3, Table S2).

### Cell surface hydrophobicity, emulsification properties, and surface charge

Cell surface charge, hydrophobicity, and emulsion stabilizing ability of the individual strains were determined on exponentially growing and stationary cells (Table S2). The results show a considerable phenotypic diversity (Figure 3, Figures S1–S3, S5–S7). The cell surface charge—measured as zeta potential—varied from −3.4 to −42.3 mV between the different strains. CSH is the measure of the extent to which cells suspended in a water phase are attracted to a hydrocarbon phase when both phases are mixed vigorously and left for phase separation to occur. We found that 25 out of the 55 strains have a CSH ranging from 0 to 20% for both growth phases. On the contrary, 9 strains showed >95% hydrophobicity for stationary growth phase, while another 5 strains showed such a high hydrophobicity in the exponential growth phase. In stationary growth phase strains NCDO712 and MG1299 displayed 60–99% hydrophobicity while the plasmid cured derivatives MG1063, MG1261, MG1362. MG1363, MG1365, and NZ9000 showed a hydrophobicity of 5–35% (Figures S5, S8, Table S2). This indicates that hydrophobicity in NCDO712 might be a plasmid-encoded trait.

Hierarchical cluster analysis revealed no correlations between the CSH of the strains as determined with different hydrocarbons and their binding of proteins (Figure 3). Strains in clade 1 show low surface hydrophobicity in the exponential growth phase, but high binding affinity to milk protein is seen. This clade consists mainly of *L. lactis* ssp *cremoris* strains of dairy origin. In contrast, strains in clade 2 poorly bind to milk proteins while they have a high CSH. Clade 3-strains show poor protein binding capacity and a low surface hydrophobicity. Clade 2 and 3 consist mainly of *L. lactis* ssp. *lactis* strains originating from the plant environment. For cells from stationary growth phase some differences are seen but the overall trends are the same in the clades 1, 3, and 4 (Figure 3B). Together, the results demonstrate that surface hydrophobicity of *L. lactis* cells and their protein binding capacity are independent parameters. Interestingly, the analysis of the origin of the strain (plant or dairy) and species of *L. lactis* revealed that the majority of *L. lactis* ssp. *cremoris* and *L. lactis* ssp. *lactis* biovar. *diacetylactis*, both of dairy origin, have a high capacity to bind to milk proteins. In contrast, the majority of strains originating from plan material only poorly bind milk proteins (Figure 3).

The stability of the emulsions obtained by mixing the water and hydrocarbon phases varied between the strains from no observed emulsification to total hydrocarbon phase emulsification and stability for at least 24 h. No correlation was found between emulsion stabilizing capacity of the strains and their cell hydrophobicity, between their charge and hydrophobicity, and between their charge/hydrophobicity and capacity to bind proteins.

A comparison of stationary-phase and exponential-phase cells revealed that 12 of the 55 strains show significant growth phase-related differences in their capacity to bind the milk protein samples tested, 3 strains show significant changes in hydrophobicity and 9 of the 55 strains show differences in the E24 measured (Figures S4, S8). The charge of stationary and exponentially growing cells differed significantly for 22 out of 55 strains tested (Figure S7). Taken together, the results indicate that the measured cell surface properties have some growth phase dependency, but strain specific properties are much more determinant. The binding of different milk proteins (regression coefficient *r* = 0.88–0.93) and cell hydrophobicity measured with different hydrocarbons (*r* = 0.98) are in relatively good agreement. However, little to no correlation is observed between hydrophobicity, cell surface charge and protein binding. Interestingly, there is a clear overrepresentation of strains of dairy origin in the clusters that show high binding ability of milk proteins which suggests that this trait might be beneficial during evolution in a dairy environment.

### Genotype-phenotype matching

A random forest-based genotype-phenotype matching algorithm (Bayjanov et al., 2013) was employed to identify genes potentially involved in cell surface properties. The analysis was run separately for each individual phenotype measured, resulting in candidate lists for genes involved in the individual traits. This resulted initially in 201 candidate genes which were selected based on the highest importance score and gene description. From these 201 candidate genes, 18 were selected for further characterization on the basis of three parameters: (i) the importance score in the individual GTM analyses; (ii) multiple appearances in the GTM analysis for the individual phenotypes; and (iii) the predicted gene function being related to cell surface (Table S3). The following choices were made for the further characterization of these genes. If the presence of a selected candidate gene was found to be associated with a phenotype and this gene is absent in *L. lactis* MG1363, it was overexpressed in MG1363. Conversely, when gene absence was found to be associated with a phenotype and this gene is present in MG1363, it was knocked-out in MG1363. We selected the longest gene within the orthologous group of the 55 strains for overexpression purposes, to eliminate the risk of working with a truncated protein. Eight of the 18 genes could not be deleted and/or overexpressed, possibly because they are essential or deleterious. Ultimately, 6 of the selected candidate genes were successfully cloned downstream of the nisin-inducible promoter P_*nisA*_ in pNZ8150 (Table 1) in *L. lactis* NZ9000, an *L. lactis* MG1363 derivative, while 4 of the genes were deleted from the chromosome of *L. lactis* MG1363 (Table 2). While we cannot exclude that the addition of nisin itself has an effect on surface properties we would like to point out that the over-expression results reported here are in relation to the nisin induced control strain which carried an empty plasmid vector. In addition no significant differences in surface properties are seen between the uninduced *L. lactis* MG1363 and its nisin induced derivative NZ9000 harboring pNZ8150 (Tables S2, S4, Table 1). Over-expression of proteins of the predicted sizes, and their cellular localization were examined with SDS-PAGE after induction with 10 ng/ml nisin of exponentially growing cells carrying the expression plasmids and incubation for another 5 h (Figure S9). The results showed that all 6 proteins could be successfully overexpressed, as they were detected either intracellularly or in the medium.

**Table 2:** Genes that were over-expressed or deleted from the chromosome.

**Gene name**	**Present in strain**	**Locus tag protein ID**	**Protein size (aa/kDa)**	**Growth phase^¥^**	**Gene presence (Pr)^¥^ Gene absence (Ab)**	**Predicted phenotype^¥^^,^^$^**	Detected phenotype^*^^,^^$^	Detected in growth phase^*^
**OVEREXPRESSION IN** ***L. lactis*** **NZ9000**								
Cell surface protein precursor	B40	B40_0084 LITC01000011 KZK48299.1	930/102.17	ST	Pr	ZP▾	CSH▴	ST
Ribose 5-phosphate isomerase A (*rpiA*)	KF147	KF147_0667 ABX75739.2	243/26.95	EX	Ab	ParaCN▴	CSH▴	ST
Internalin, putative (LPXTG motif)	KF282	KF282_0409 -	559/61.28	ST	Pr	ZP▴	CSH▴	EX, ST
Hypothetical protein (*yreB*)	IL1403	L128699 AAK05785.1	314/35.64	EX	Pr	CSH▴	None	–
Cell wall surface anchor family protein	KF147	LLKF_0311 ADA64081	809/87.46	EX EX	Pr Ab	CSH▴ E24▴	CSH▴ ParaCN▾ NaCN▾ NaCN90C▾	EX, ST ST ST ST
Endo-beta-N-acetylglucosaminidase (*ypcCD*)	KF147	LLKF_1605 ADA65249	923/102.53	ST ST	Ab Ab	ParaCN▴ NaCN▴	CSH▴	ST
**KNOCKOUT IN** ***L. lactis*** **MG1363**								
Conjugal transfer protein (*traG*)	MG1363	llmg_1383 CAL97970	612	EX	Ab	ParaCN▾	ParaCN▾ NaCN▾ NaCN90C▾	EX EX EX
Cell surface protein precursor	MG1363	llmg_1096 CAL97690	387	ST	Ab	ZP▾	No	–
Hypothetical protein	MG1363	llmg_1093 CAL97687.1	334	ST	Pr	NaCN▴	NaCN▾	EX, ST
Sortase SrtA (*srtA*)	MG1363	llmg_1449	250	–	–	–	No	–

¥ *These columns indicate if either the presence or absence of a particular gene of cells from exponential (EX) or stationary (ST) growth phase resulted in an altered phenotype*.

* *These columns indicate phenotypic changes that were detected in a particular growth phase in engineered strains where the indicted genes were either overexpressed or deleted*.

$ *ZP, charge (mV); ParaCN, attachment to paracaseinate (%); NaCN, attachment to sodium caseinate (%); NaCN90C, attachment to sodium caseinate heated at 90°C for 10 min (%); CSH, cell surface hydrophobicity (%); E24, emulsion stability for 24 h (%); ▴, cell surface property increases; ▾, cell surface property decreases. All phenotype changes indicated are significant with p < 0.01*.

### Cell surface properties of the recombinant *L. lactis* strains

The deletion of the gene *llmg_1383* decreased the binding of exponentially growing cells of MG1363 to the 3 milk proteins tested by 53 ± 3%. The protein Llmg_1383 of *L. lactis* MG1363 is annotated as a conjugal transfer protein TraG, which is described to aid in the transfer of DNA during bacterial conjugation. As it is predicted to be involved in membrane pore formation (type IV secretion; Schroder et al., 2002), a role in surface alteration seems plausible.

Similar to *llmg_1383*, the deletion of the hypothetical gene *llmg_1093* also decreased the binding of *L. lactis* MG1363 to the three milk proteins examined by 60 ± 27% for cells in the exponential growth phase and by 42 ± 37% for cells in the stationary growth phase. Protein Llmg_1093 is a putative secreted protein but its actual function is not known.

As several of the genes studied here harbor sortase dependent LPXTG signals we also deleted the sortase A gene (*llmg_1449)* from the chromosome of strain *L. lactis* MG1363. This deletion did not change the cell surface properties of *L. lactis* MG1363 (Table S4), indicating that SrtA might not be involved directly in cell surface properties. We also tried to obtain a knock-out mutant of the *srtC* gene but were not able to obtain it after two attempts, indicating a possibly essential role or SrtC for growth.

The cell surface protein precursor B40_0084 is a putative mucus binding protein as it has 4 mucus binding domains (MucBP) (pfam06458) and a LPXTG-motif cell wall anchor domain (TIGR01167). GTM predicted that the presence of this protein leads to a more negative cell surface charge. This prediction was not confirmed, but changes were observed for hydrophobicity. For example, for stationary phase cells where the control strain shows a CSH of 8.8% (±5%), the overexpression of B40_0084 resulted in a CSH of 89% (±7%) (Table S4). Interestingly, while SDS PAGE analysis (Figure S9) verified the overexpression of B40_0084 in exponentially growing cells, this overexpression did not affect the CSH of the cells in this growth phase. Mucus binding proteins are described to be involved in the binding of carbohydrates such as mannose (Pretzer et al., 2005) and they are speculated to be important for probiotic function (Kleerebezem et al., 2010). The effect of the overexpression of *B40_0084* only leads to hydrophobicity changes in stationary phase cells, indicating that other surface decoration(s) dominates surface properties in a growth phase-dependent manner.

Another gene identified by gene-trait matching is a putative internalin containing four MucBP domains (pfam06458) and a surface-anchoring domain (COG4932). Internalins were originally described in *Listeria monocytogenes* as surface proteins that are involved in adhesion to mammalian epithelial cells (Lecuit et al., 1997). For example, protein internalin A (InlA) mediates bacterial adhesion and invasion of epithelial cells in the human intestine through specific interaction with its host cell receptor E-cadherin (Schubert et al., 2002). Here, we overexpressed the ortholog from strain KF282 (KF282_0409). While gene-trait matching associated the presence this gene with lower surface charge, its overexpression in MG1363 increased the CSH, from 8.8% (±5) in the control strain to 49.3% (±21.8). Furthermore, overexpression of a cell wall surface anchor family protein (LLKF_0311) led to the decreased attachment of cells to milk proteins from 98% (±0.7) to about 31% (±47%) in the stationary growth phase and to the increase in CSH from 9 ± 5 to 78 ± 9% in the stationary and exponential growth phases (Table S4) while initially the presence of *LLKF_0311* was predicted to effect cells surface hydrophobicity and emulsion stability in exponential phase of growth. (Table S4).

The endo-beta-N-acetylglucosaminidase *ypcCD* (*LLKF_1605)* was found to be associated to the binding of cells to milk proteins. Overexpression of this protein did not lead to an alteration in attachment of cells in stationary growth phase to milk proteins, but led to an increase in surface hydrophobicity from about 8.8 ± 5 to 23.7 ± 16.1%.

Overall, the phenotype predicted by gene-trait matching could be confirmed experimentally for 3 out of 10 engineered strains, and for 2 out of 3 strains additional altered surface properties were detected. Overexpression of the selected candidate genes did not influence the protein binding properties. A total of 4 strains showed altered surface properties but not the predicted ones whereas for 3 strains no changes were observed. While not all predictions were correct, the approach did allow identifying targets that are of importance for lactococcal surface properties.

## Discussion

This study describes bacterial surface properties such as cell surface charge, hydrophobicity and the attachment to milk proteins for 55 *L. lactis* strains isolated from either plant material or the dairy environment. A flow cytometry based method for the characterization of protein binding allowed to demonstrate the existence of a large biodiversity in cell attachment to milk proteins. We show that the capacity of cells to bind the milk proteins is growth phase-dependent for some of the strains tested. Importantly, this methodology is not restricted to the use of milk proteins and we expect it to be applicable for the characterization of cell attachment to other proteins, and for other bacterial species. In combination with cell sorting the method may prove useful in enabling the selection of cells with desired surface characteristics.

In contrast to a previous executed GTM study with *L. lactis* strains, which was done based on comparative genome hybridization data (Bayjanov et al., 2013), we were able to use either draft or complete genome sequences, which should increase the predictive power of the approach. The characterization of selected target genes, by their overexpression and/or deletion from the chromosome, resulted in the identification of 7 genes that are involved in cell surface properties.

The observed biodiversity of cells obtained from different growth phases might be explained by differences in the molecular composition of the cell wall. For example, peptidoglycan modification during exponential growth in *L. lactis* results only in partial (75%) amidation of the alpha-carboxyl group of the D-Asp cross-bridge to the PG precursor (Courtin et al., 2006; Veiga et al., 2009). In contrast, the amidation of amino acids during peptidoglycan modification for *L. casei* is almost complete (near 100%) during both growth phases (Chapot-Chartier and Kulakauskas, 2014). However, peptidoglycan is not the major component exposed at the bacterial surface, but it is rather dominated by polysaccharides, teichoic acids, and proteins. The charge of the cell surface is mainly determined by net charge of molecular composition of cell wall. For example, a negative cell surface charge can be partially determined by carboxyl and phosphate groups of LTA and TA, while positive charges partially derive from D-alanine molecules that are esterified to TA and LTA (Delcour et al., 1999).

Overall we see poor or no correlations between properties such as emulsion stabilizing ability and cell hydrophobicity, between charge and hydrophobicity, or between charge/hydrophobicity and the attachment to proteins. This might be caused by the amphiphilic surface properties of bacteria (Van Oss, 2003).

While there is ample literature describing the cell surface and cell wall composition of bacteria in general, available information on key molecules determining cell surface properties of lactic acid bacteria are limited. For instance, the overexpression of the surface anchored protein CwaA from *Lactobacillus plantarum* NL42 in *L. lactis* NZ9000 led to cell auto-aggregation, increased hydrophobicity and attachment of the CwaA-producing *L. lactis* cells to human epithelial HT-29 cells (Zhang et al., 2015). Other studies describe the autolysins AcmA and AcmD, which are involved in cell chaining (Visweswaran et al., 2013), or the expression of pili on the surface of *L. lactis*. Pili can be plasmid- as well as genome-encoded and they have been shown to cause auto-aggregation (Oxaran et al., 2012; Tarazanova et al., 2016) and to increase attachment to epithelial cells (Meyrand et al., 2013; Zhang et al., 2015). The *L. lactis* cell wall proteinase PrtP was also shown to be involved in cell surface properties and adhesion to solid surfaces (Habimana et al., 2007).

We successfully verified the influence of 3 proteins on predictions based on the performed gene-trait matching. However, the alteration of protein expression in some of our engineered strains did result in an effect on the cell surface other than the predicted one. The underlying molecular details of these discrepancies are not clear, but we speculate that affected molecules are in competition for space on the cell surface. Alteration of the expression level of one molecule would indirectly affect the overall surface composition, which could result in unexpected phenotypic outcomes. Such a speculation is in line with recent theory on trade-offs that can be determined by physical-chemical constraints such as membrane space (Bachmann et al., 2016, 2017). While most of the genes identified here could be linked to the cell surface we could not find such a link for some identified proteins based on sequence analysis. The over-expression of a ribose-5-phosphate isomerase, an enzyme involved in the pentose phosphate pathway, altered CSH. A direct role of this enzyme in cell surface properties seems unlikely, but a study in *L. plantarum* suggests that ribose acts as a precursor for alternative cell wall teichoic acids (Bron et al., 2012) and it is therefore conceivable that a change in ribose availability could lead to altered cell wall properties. The identification of such proteins, that cannot be linked to cell surface properties with e.g. sequence based motif analysis, points out the added predictive value of genotype/phenotype matching.

An interesting observation is the fact that strains isolated from a dairy environment show much stronger binding of milk proteins as compared to plant isolates. Literature holds many examples for the role of surface alterations to improve the fitness of an organism in a particular environment. In pathogenic bacteria, for instance, peptidoglycan modifications allow escaping the host's immune system (Foster, 2005) and in soils the capacity to form biofilms is a key factor for microbial fitness (Nazir et al., 2010). The proposed evolutionary transition of *L. lactis* from the plant to the dairy environment is described to be accompanied by the loss of the ability to synthesize several amino acids or to catabolize typical plant-derived sugars. The occurrence of amino acid auxotrophies in dairy isolates is compensated by improved utilization of milk proteins through e.g. extracellular proteases, dedicated (oligo)peptide transport systems and intracellular peptidases (van Hylckama Vlieg et al., 2006; Bachmann et al., 2012). The fact that binding to milk proteins was selected for in a dairy environment suggests a selective advantage, which seems plausible seen the growth dependency of dairy strains of *L. lactis* on extracellular amino acids. It will be interesting to see if the alteration of surface properties of *L. lactis* also impacts on the functionality of starter cultures in pure and mixed-culture fermentations.

## Author contributions

MT, TH, JK, HB conceived the research. MT, MB, SvS, PJ carried out the experiments. MT, MW, HB analyzed the data. MT, JK, HB wrote the manuscript. All authors commented on the manuscript.

### Conflict of interest statement

The authors declare that the research was conducted in the absence of any commercial or financial relationships that could be construed as a potential conflict of interest.
