# Supplementary material for: Cell Surface Properties of Lactococcus lactis Reveal Milk Protein Binding Specifically Evolved in Dairy Isolates
Source: Front Microbiol. 2017 Sep 7;8:1691. doi: 10.3389/fmicb.2017.01691 (PMC5594101; doi:10.3389/fmicb.2017.01691)
Supplement: Supplementary file 1 [file DataSheet1.docx]

**Supporting Information**

**Cell surface properties of *Lactococcus lactis* reveal milk protein binding specifically evolved in dairy isolates**

Authors: Mariya Tarazanova^1,2,3^, Thom Huppertz^1,2^, Marke Beerthuyzen^1,2^, Saskia van Schalkwijk^1,2^, Patrick Janssen^1,2^, Michiel Wels^1,2^, Jan Kok^2,3^, Herwig Bachmann^1,2^

^1^ NIZO B.V., P.O. Box 20, 6710 BA, Ede, The Netherlands

^2^ TI Food and Nutrition, P.O. Box 557, 6700 AN Wageningen, The Netherlands

^3^ Molecular Genetics, University of Groningen, Nijenborgh 7, 9747AG, Groningen, The Netherlands

Corresponding Author: [Herwig.Bachmann@nizo.com](mailto:Herwig.Bachmann@nizo.com)


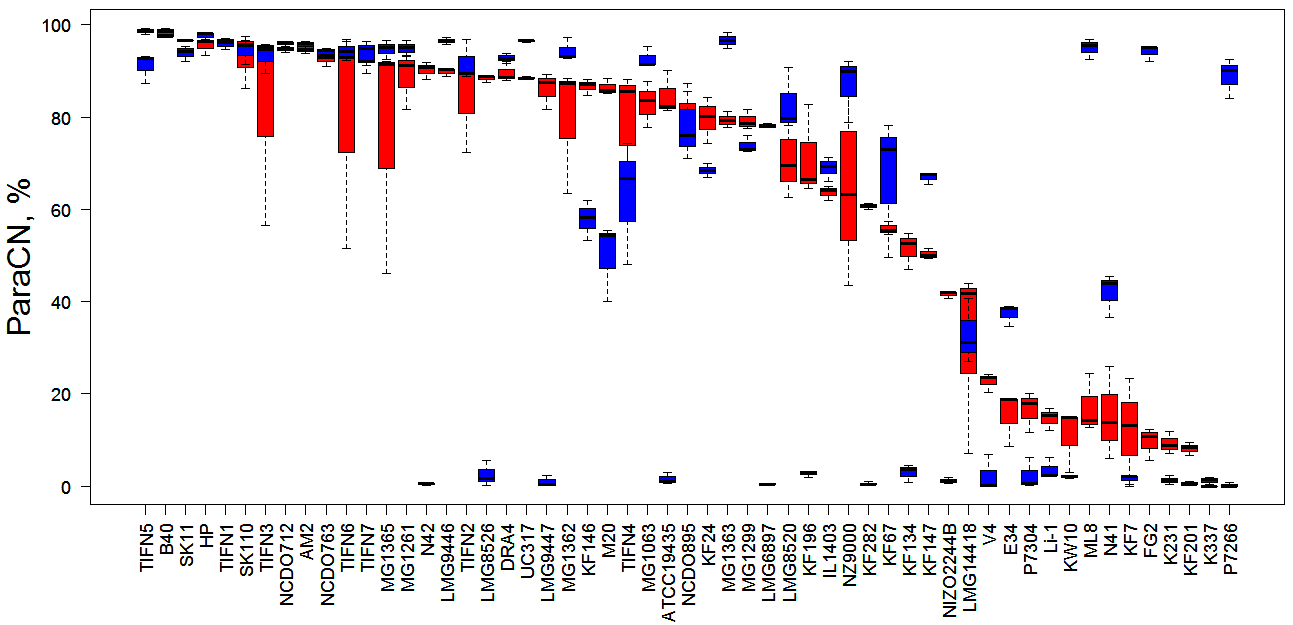


**Figure S 1.** Attachment of para-caseinate (ParaCN, %) (y-axis) to 55 *Lactococcus lactis* strains (x-axis) (n=3)*.* Bacterial strains were sorted from the highest to the lowest value for cells from stationary growth phase (shown in red). The values measured for cells from exponential growth phase are shown in blue.


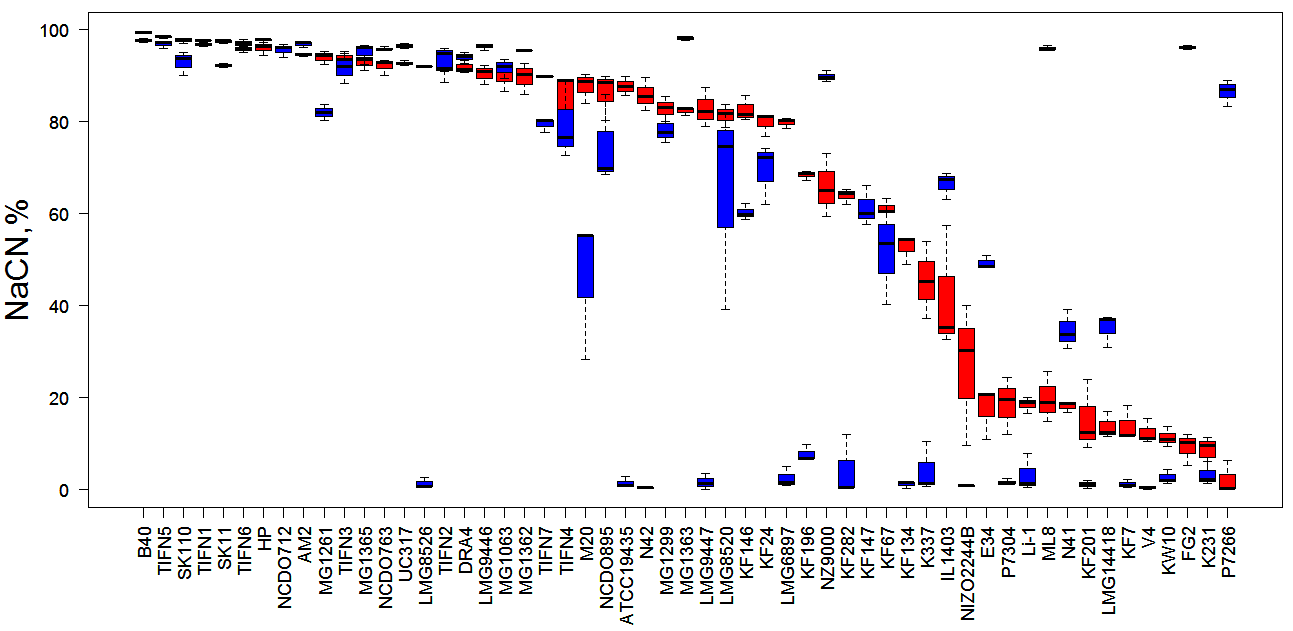


**Figure S 2.** Attachment of sodium caseinate (NaCN, %) (y-axis) to 55 *Lactococcus lactis* strains (x-axis) (n=3). Bacterial strains were sorted from the highest to the lowest value for cells from stationary growth phase (shown in red). The values measured for cells from exponential growth phase are shown in blue.


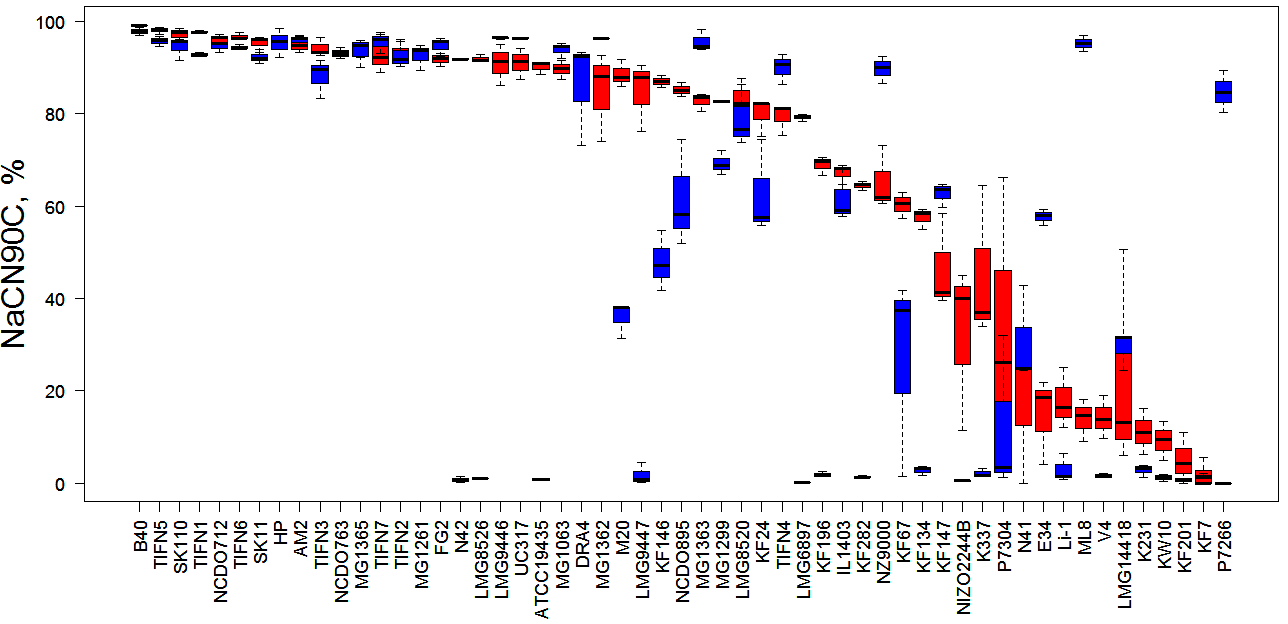


**Figure S 3.** Attachment of sodium caseinate heated for 10 min at 90°C (NaCN90C, %) (y-axis) to 55 *Lactococcus lactis* strains (x-axis) (n=3). Bacterial strains were sorted from the highest to the lowest value for cells from stationary growth phase (shown in red). The values measured for cells from exponential growth phase are shown in blue.


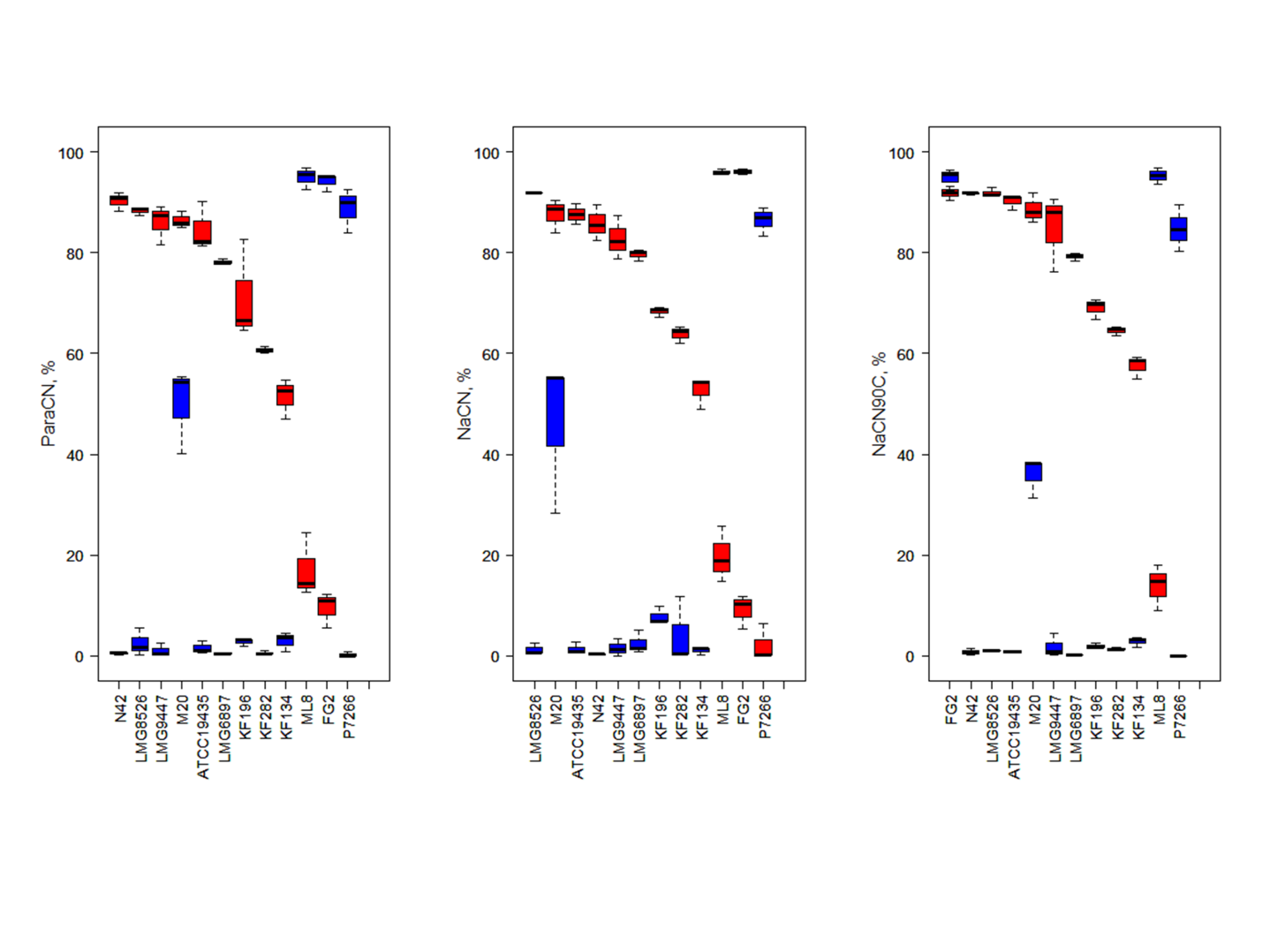


**Figure S 4.** Twelve out of 55 *Lactococcus lactis* strains (x-axis) which showed opposite attachment behavior to milk proteins (y-axis) when either originating from exponential or stationary growth phases, (n=3): ParaCN,% indicates attachment to para-caseinate, NaCN,% - attachment to sodium caseinate, and NaCN90C,% - attachment to sodium caseinate heated for 10 min at 90°C. Bacterial strains were sorted from the highest to the lowest value for stationary growth phase (shown in red). The values measured at exponential growth phase are shown in blue.


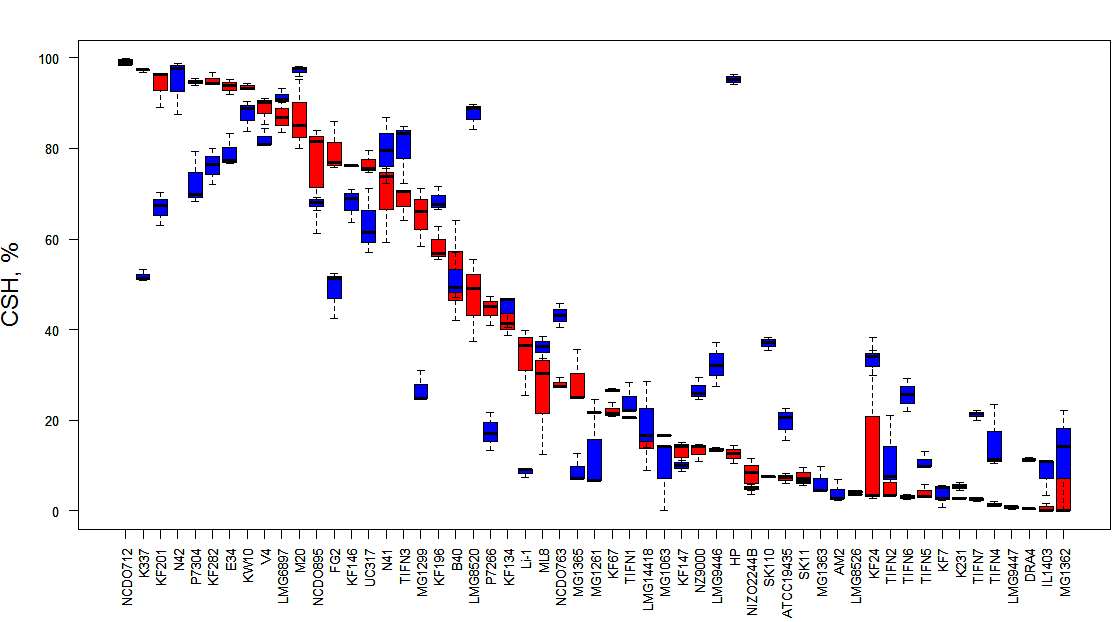


**Figure S 5.** Hydrophobicity (CSH, %) (y-axis) of 55 *Lactococcus lactis* strains (x-axis) (n=3)*.* Bacterial strains were sorted from the highest to the lowest value for cells from stationary growth phase (shown in red). The values measured for cells from exponential growth phase are shown in blue.


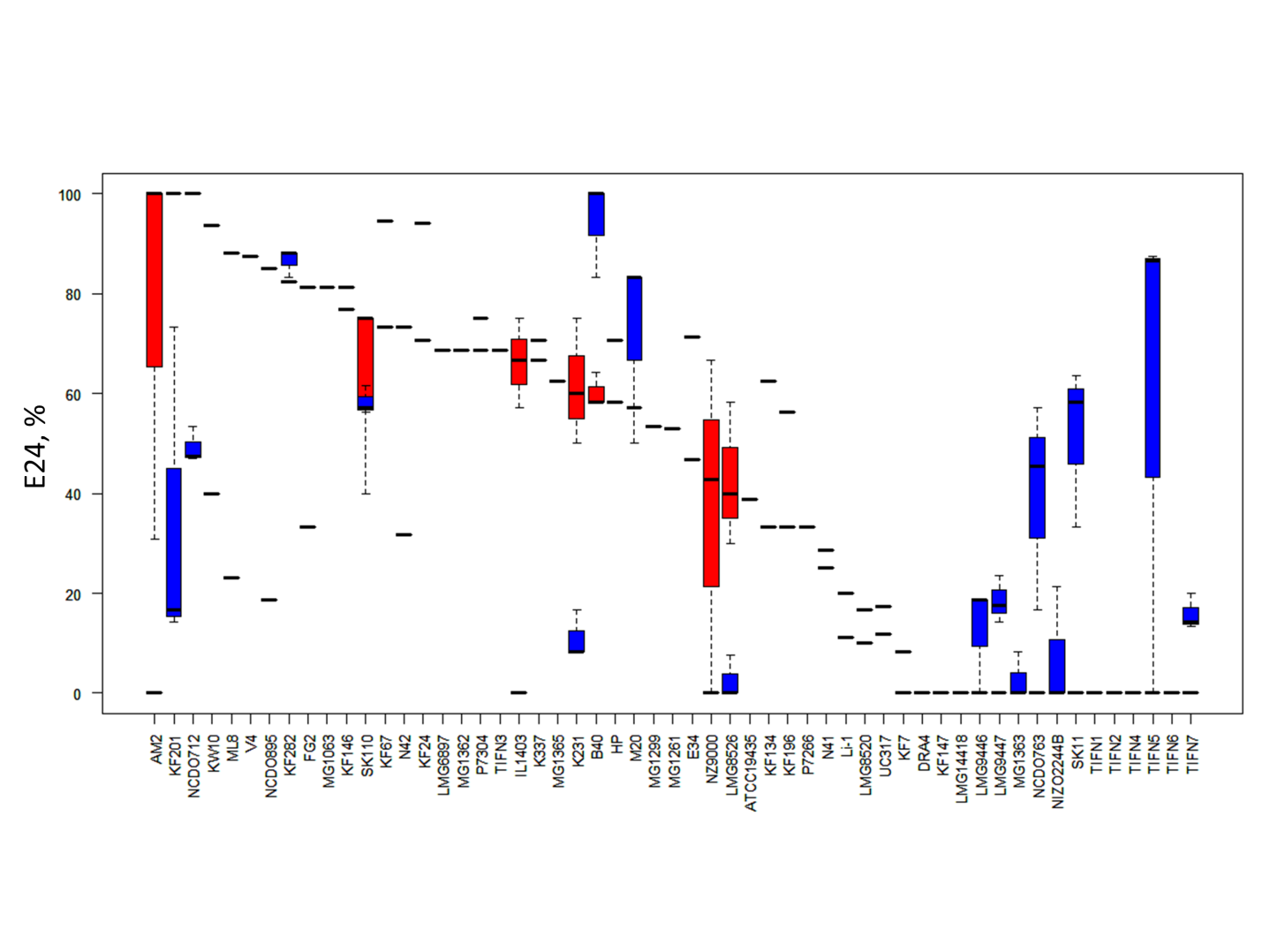
 **Figure S 6**. Emulsion stability (E24, %) (y-axis) of 55 *Lactococcus lactis* (x-axis) (n=3). Bacterial strains were sorted from the highest to the lowest value for cells from stationary growth phase (shown in red). The values measured for cells from exponential growth phase are shown in blue.


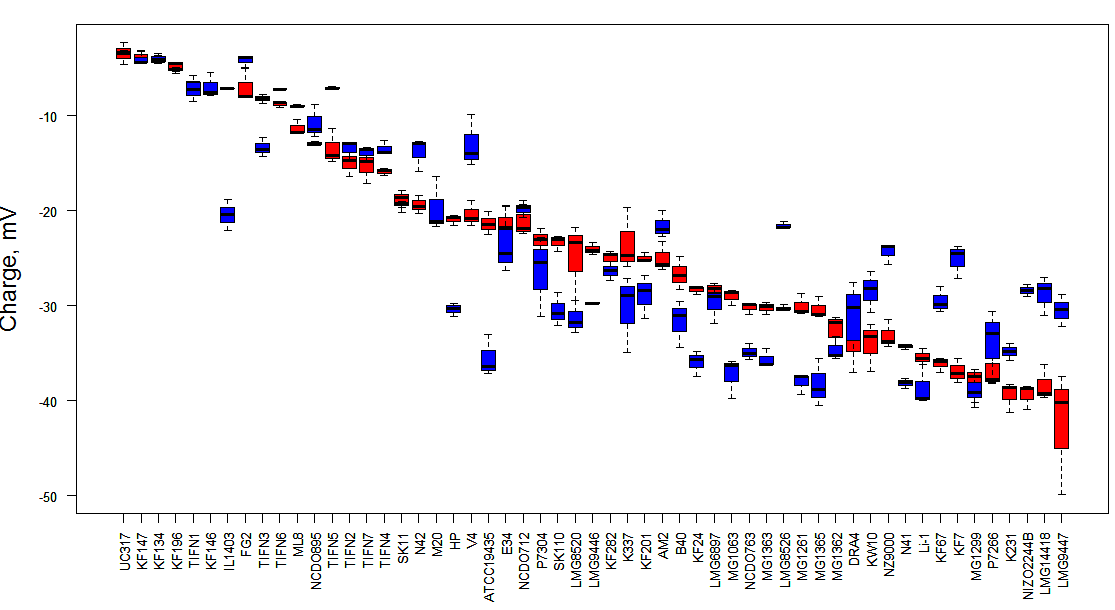


**Figure S 7.** Charge (mV) (y-axis) of 55 *Lactococcus lactis* (x-axis) (n=3)*.* Bacterial strains were sorted from the highest to the lowest value for cells from stationary growth phase (shown in red). The values measured for cells from exponential growth phase are shown in blue.


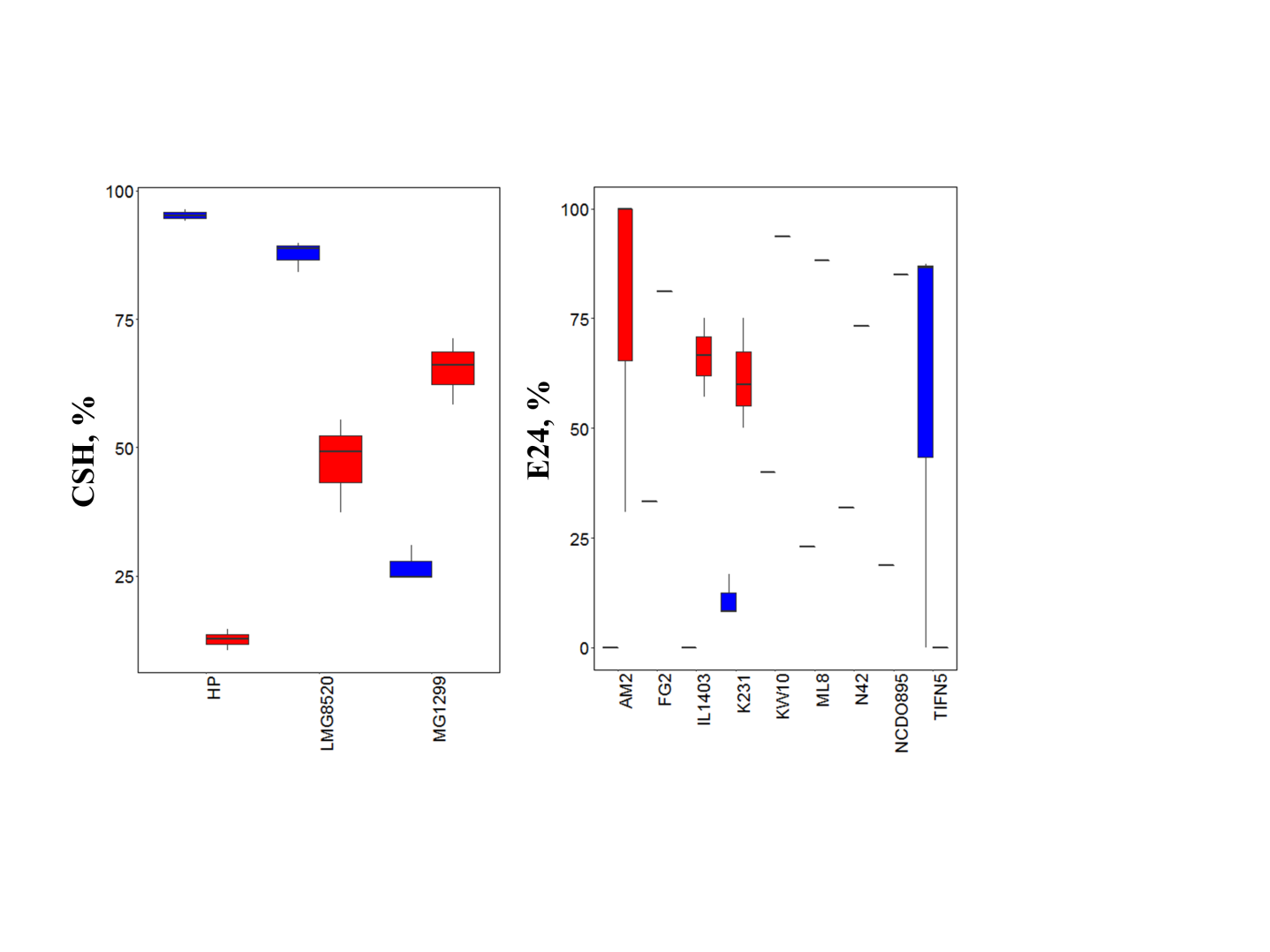
 **Figure S 8.** *Lactococcus lactis* (x-axis) with opposite cell surface hydrophobicity (CSH, %) (y-axis) and emulsion stability (E24,%) (y-axis) when originating either from exponentially growing (blue) or stationary cultures (red) (n=3).


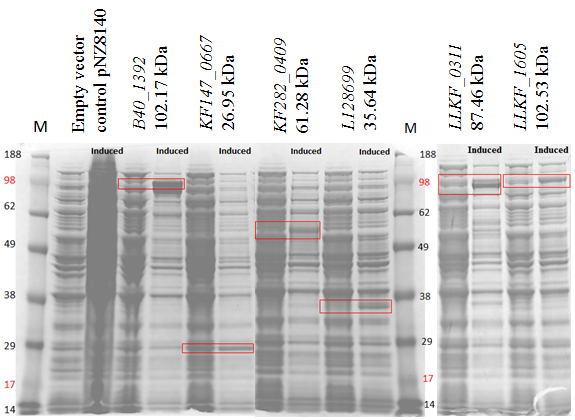


A


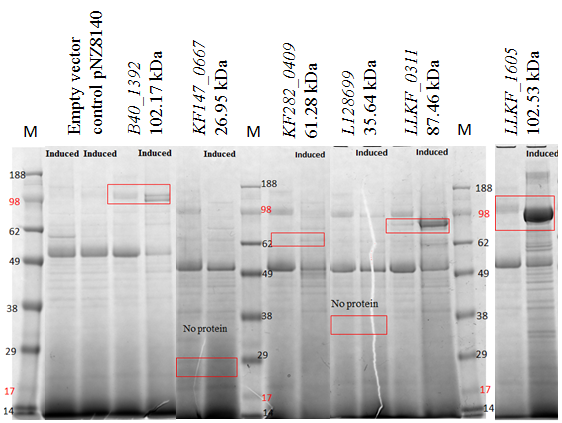


B

**Figure S 9.** SDS-PAGE gels of the 6 overexpressed proteins in *L. lactis* NZ9000 after induction of exponentially growing cells with 10 ng/ml nisin and incubation for another 5 h. Overexpressed proteins were isolated either from cells (Panel A) or from supernatant (Panel B). Bands of overexpressed proteins are indicated in red boxes. M – marker, λ-HindIII.

**Table S 1. Oligonucleotides used in this study.**

| **Name ^a^** | **Sequence (5’ to 3’)** |
| --- | --- |
| 1. **Primers used for gene over-expression** | |
| OG3116 forw | ATGATAGCGAAGAACCCTAACACACCAGC |
| OG3116 revXbaI | GGCTCTAGATAATCACGTTTAGGCTTGCGTTTAACG |
| OG2970forw | ATGACGAAAGAAAATATCGCGAACTTGGC |
| OG2970revXbaI | CCGTCTAGACTACAATTCCCCAATTATATTTTCCCATTC |
| OG2478forw | ATGAATAAGAAAACCCTTATCTCGTCAC |
| OG2478revXbaI | CCGTCTAGAGTTTCCACATTTTCCATAAATCTATTATATCG |
| OG1984forw | ATGCGTAAATTGGAAAAATTGTTGAATGACTTGACG |
| OG1984revXbaI | CCGTCTAGACAAGTCTGCTCTTTTCTATCCTTCTTC |
| OG1874forw | ATGAAAAAATCGAAAAAATTTTTTATTAGAATATGCCTTTCTACAGGAATTTTGGC |
| OG1874revXbaI | CCGTCTAGAGGCAAACTTTTACAGCCCTTTGTCAGC |
| OG2225forw | ATGAAAAATAAAATTATCGTAGTAGCTATACTAATAACAAC |
| OG2225revXbaI | GGCTCTAGATTAATTTGTGGTTTCTACGGGGCTTAAATAAGC |
| 1. **Oligonucleotides for amplification of sequences used to generate double cross-over mutants** | |
| OG2161LFforwXbaI | CCGTCTAGAGAGTACGAACGTCAAACTGAGCAGC |
| OG2161LFrev + vlag | GCTCTAGGATGTTCATAGGCATAATATTTGCCCAGGTAACATCCAGAAGAAATTACCTAT |
| OG2161RFforw | GCAAATATTATGCCTATGAACATCCTAGAGC |
| OG2161RFrevEcoRI | GGCGAATTCGGCTCGATAACCATAAGTTGGATTCCC |
| OG2161DCOforw | GGACGAAGTGGGTTTGCCTTTTGC |
| OG2161DCOrev | GTTGAGCTTGTTGGCTATTGAGTGC |
| OG1903LFforwXbaI | CCGTCTAGACGTCATTAGCACAGTCTTACTAGCAACC |
| OG1903LFrev + vlag | GACCTAAGATGAAGAGCAACCAAGTCCGGTTTGACTAAAAGCAACTTGTGCACCTCC |
| OG1903RFforw | GGACTTGGTTGCTCTTCATCTTAGGTC |
| OG1903RFrevSacI | GGCGAGCTCGAGAGCTTCCTTACTTTAGCTGATGG |
| OG1903DCOforw | CGGCAACTAATCCACTCACACCTGG |
| OG1903DCOrev | CCAGCTCGTGACCTTGGTCCTCGC |
| OG1859LFforwXbaI | GCCTCTAGAGGTTTCGCTTGCTCCAGGATTAGG |
| OG1859LFrev + vlag | CGCTCCAGATACAATAAAAACAAGAATGACCCCTTCAAGTACCGCTTCTATACTAAAATCACC |
| OG1859RFforw | GGTCATTCTTGTTTTTATTGTATCTGGAGCG |
| OG1859RFrevEcoRI | CCGGAATTCGCAGGTTTGTTCCTGTTCCCGTTCCTGC |
| OG1859DCOforw | GACCCAACGACTAAAGCGCAGACAC |
| OG1859DCOrev | GAGTGCCAGCAATGTCAAAGTATCACC |
| srtALFforwBamHI | CCCGGATCCGGATAAAGAAGCAGCAGTTGATAAAGCC |
| srtALFrev + vlag | CACAGAATCTGTCACTTTATCAAATGCCTTCTCGCGCTCTTCCATCTATATATGACCACC |
| srtARFforw | GAGAAGGCATTTGATAAAGTGACAGATTCTGTG |
| srtARFrevEcoRI | GGCGAATTCCGCAAAATAAGCAAGAGAATCTTCATCTCC |
| srtADCOforw | GGTGACGGTTTATCAACCATGATTTATGC |
| srtADCOrev | CCAAAAGCTTGGCCGCAGTTTCTTGCC |
| **^a^** The letters “rev” and “forw” indicate oligonucleotides annealing to the DNA strand in reverse or forward direction, respectively. XbaI, EcoRI, SacI, BamHI indicate restriction sites within the sequence. LF and RF indicates if the oligonucleotide is located upstream or downstream of the amplified gene and the annotation +vlag indicates nucleotides in a “LF” amplicon that overlaps with the “RF” amplicon which allows to perform a SOE-PCR. The letters DCO indicate oligonucleotides used to confirm double crossover mutants. | |

**Table S 2. Cell surface properties of 55 lactic acid bacteria (n=3).** Surface properties were measured at exponential and stationary growth phases. We measured the cell surface hydrophobicity (CSH, %) to two hydrocarbons – petroleum (PCSH, %) and hexane (HCSH, %), emulsion stability (E24, %), charge (ZP, mV), attachment to milk proteins: para-caseinate (ParaCN, %), sodium caseinate (NaCN, %) and sodium caseinate heated for 10 min at 90°C (NaCN90C, %). The values represent the (average ± standard deviation) calculated for 3 biological replications.

| **Strain** | **Growth phase** | **ParaCN, %** | **NaCN, %** | **NaCN90C, %** | **PCSH, %** | **E24P, %** | **HCSH, %** | **E24H, %** | **ZP, mV** |
| --- | --- | --- | --- | --- | --- | --- | --- | --- | --- |
| AM2 | Exponential | 95.7±0.9 | 96.8±0.6 | 95.9±1.2 | 4.1±2.4 | 0±0 | 2.3±0.6 | 0±0 | -21.6±1.4 |
| ATCC19435 | Exponential | 1.5±1.3 | 1.4±1.2 | 0.8±0.3 | 19.6±3.7 | 38.9±0 | 22±1.4 | 35±0 | -35.5±2.2 |
| B40 | Exponential | 97.5±0.2 | 97.7±0.4 | 97.9±0.8 | 51.2±5.2 | 94.4±9.6 | 52.3±3.7 | 94.4±9.6 | -31.6±2.5 |
| DRA4 | Exponential | 92.8±1.1 | 93.9±1.3 | 86.3±11.4 | 11.4±0.4 | 0±0 | 12.3±1.5 | 0±0 | -31.6±4.9 |
| E34 | Exponential | 37.3±2.4 | 49.1±1.5 | 57.7±1.7 | 79.1±3.6 | 71.4±0 | 86.8±1 | 56.3±0 | -23.4±3.6 |
| FG2 | Exponential | 94.2±1.7 | 96.1±0.5 | 94.8±2.1 | 48.7±5.4 | 33.3±0 | 71.1±4 | 38.5±0 | -4.2±0.7 |
| HP | Exponential | 97.5±1 | 97.7±0.4 | 95.4±3.2 | 95.2±1.1 | 70.6±0 | 94.6±0.9 | 70.6±0 | -30.4±0.7 |
| IL1403 | Exponential | 68.9±2.6 | 66.3±3 | 61.7±5.6 | 8.4±4.3 | 0±0 | 2.8±1.4 | 0±0 | -20.4±1.7 |
| K231 | Exponential | 1.3±0.9 | 3.1±2.6 | 2.8±1.3 | 5.4±0.9 | 11.1±4.8 | 4.6±1.4 | 11.1±4.8 | -34.8±0.9 |
| K337 | Exponential | 1.3±0.8 | 4.1±5.4 | 2.1±1 | 51.8±1.2 | 70.6±0 | 54.4±2.4 | 62.5±0 | -30.3±4.1 |
| KF134 | Exponential | 3±1.9 | 1.1±0.9 | 2.8±1 | 44.7±3.6 | 62.5±0 | 46.2±6.9 | 41.2±0 | -4±0.5 |
| KF146 | Exponential | 57.9±4.4 | 60.2±1.7 | 47.9±6.5 | 67.9±3.8 | 81.3±0 | 71.1±3.1 | 81.3±0 | -7±1.3 |
| KF147 | Exponential | 66.9±1.3 | 61.2±4.3 | 62.7±2.7 | 10±1.2 | 0±0 | 7.3±1.3 | 0±0 | -4±0.6 |
| KF196 | Exponential | 2.7±0.7 | 7.8±1.7 | 1.9±0.5 | 68.5±2.7 | 56.3±0 | 51.6±5 | 58.8±0 | -5.2±0.2 |
| KF201 | Exponential | 0.6±0.4 | 1.1±0.9 | 0.6±0.5 | 66.8±3.7 | 34.8±33.4 | 72.4±2.2 | 34.8±33.4 | -28.8±2.3 |
| KF24 | Exponential | 68.4±1.5 | 69.4±6.6 | 62.6±10.2 | 33.1±2.8 | 94.1±0 | 27.3±8.4 | 94.1±0 | -35.9±1.3 |
| KF282 | Exponential | 0.6±0.3 | 4.1±6.7 | 1.4±0.3 | 76.2±4.1 | 86.6±2.8 | 71.7±0.8 | 86.6±2.8 | -26.3±1 |
| KF67 | Exponential | 66.9±15.2 | 51.8±10.9 | 26.8±22.2 | 26.6±0.4 | 94.4±0 | 27.4±0.9 | 94.4±0 | -29.5±1.3 |
| KF7 | Exponential | 1.6±1 | 1.1±0.9 | 1.4±0.5 | 3.8±2.7 | 0±0 | 4.6±0.2 | 0±0 | -25.1±1.8 |
| KW10 | Exponential | 2.1±0.3 | 2.5±1.6 | 1.2±0.8 | 87.6±3.4 | 40±0 | 82±7.4 | 17.6±0 | -28.4±2.2 |
| Li-1 | Exponential | 3.6±2.3 | 3.1±4 | 2.9±3.1 | 8.6±1.1 | 11.1±0 | 9.7±2.8 | 10.5±0 | -38.6±2.1 |
| LMD9 | Exponential | 93.3±2.9 | 91.5±3.2 | 86.2±4.3 | 45±4.8 | 53.5±34.3 | 42.3±3.4 | 53.5±34.3 | -4.6±1.1 |
| LMG14418 | Exponential | 33±7 | 35±3.6 | 29.2±4.2 | 19.8±7.7 | 0±0 | 12.8±1.9 | 0±0 | -28.7±2.1 |
| LMG6897 | Exponential | 0.4±0.2 | 2.4±2.3 | 0.2±0.1 | 91.4±1.6 | 68.8±0 | 93.4±1.7 | 68.8±0 | -29.7±1.9 |
| LMG8520 | Exponential | 82.8±6.9 | 65.1±22.7 | 78.9±6.6 | 87.6±3 | 10±0 | 95.2±0.8 | 9.1±0 | -31.3±1.7 |
| LMG8526 | Exponential | 2.5±2.8 | 1.2±1.2 | 1.1±0.1 | 4.2±0.2 | 2.6±4.4 | 4.1±0.9 | 2.6±4.4 | -21.6±0.4 |
| LMG9446 | Exponential | 96.5±0.8 | 96.3±0.7 | 96.4±0.3 | 32.3±4.9 | 12.5±10.8 | 24.9±1 | 12.5±10.8 | -29.7±0.1 |
| LMG9447 | Exponential | 1±1.3 | 1.6±1.8 | 1.8±2.4 | 0.8±0.5 | 18.5±4.7 | 0.6±0.2 | 18.5±4.7 | -30.5±1.7 |
| M20 | Exponential | 49.9±8.5 | 46.2±15.6 | 35.9±3.9 | 97.2±1.1 | 72.2±19.2 | 93.8±2.7 | 78.6±0 | -19.7±2.9 |
| MG1063 | Exponential | 92.6±2.3 | 91.6±2.2 | 93.9±1.8 | 9.5±8.2 | 81.3±0 | 8.8±1.3 | 62.5±0 | -37.3±2.1 |
| MG1261 | Exponential | 94.9±1.7 | 82±1.6 | 92.6±2.8 | 12.7±10.3 | 52.9±0 | 12.1±7.2 | 46.7±0 | -38±1.1 |
| MG1299 | Exponential | 73.9±1.8 | 78.2±3.1 | 69.2±2.6 | 26.8±3.6 | 53.3±0 | 32.4±5.9 | 40±0 | -38.8±1.6 |
| MG1362 | Exponential | 94.4±2.5 | 95.4±0.1 | 96.3±0.2 | 12.2±11.3 | 68.8±0 | 14.7±14 | 50±0 | -34.6±1.3 |
| MG1363 | Exponential | 96.6±1.7 | 98±0.3 | 95.6±2.4 | 6.3±3.1 | 2.8±4.8 | 3.6±0 | 2.8±4.8 | -35.6±1 |
| MG1365 | Exponential | 94.7±2.1 | 95±2.2 | 93.6±3.1 | 9±3.1 | 62.5±0 | 10.2±1.5 | 43.8±0 | -38.3±2.5 |
| ML8 | Exponential | 94.9±2.3 | 95.9±0.5 | 95.3±1.6 | 36.1±2.4 | 23.1±0 | 45.3±3.8 | 23.1±0 | -8.9±0.1 |
| N41 | Exponential | 42±4.7 | 34.5±4.3 | 30.7±10.5 | 79.6±7.3 | 25±0 | 90.4±3.4 | 21.4±0 | -38.1±0.6 |
| N42 | Exponential | 0.4±0.3 | 0.3±0.1 | 0.8±0.7 | 94.7±6.2 | 31.8±0 | 97.8±1.4 | 30±0 | -13.8±1.8 |
| NCDO712 | Exponential | 95.9±0.6 | 95.4±1.3 | 94.6±1.2 | 98.7±0.5 | 49.3±3.5 | 98.8±0.8 | 50.1±7.7 | -19.9±0.7 |
| NCDO763 | Exponential | 94.1±1 | 95.8±0.3 | 93.3±0.9 | 43.1±2.6 | 39.8±20.8 | 29.6±3 | 39.8±20.8 | -34.9±0.8 |
| NCDO895 | Exponential | 78±8 | 75±10 | 62±12 | 68±1.4 | 19±0 | 82±0.4 | 22±0.2 | -11±1.8 |
| NIZO2244B | Exponential | 1.2±0.7 | 0.8±0.2 | 0.5±0.2 | 5.1±0.6 | 7.1±12.4 | 4±1.3 | 7.1±12.4 | -28.4±0.7 |
| NZ9000 | Exponential | 87±7.1 | 89.8±1.2 | 89.7±3 | 26.6±2.5 | 0±0 | 25.7±4.8 | 0±0 | -24.3±1.1 |
| P7266 | Exponential | 88.8±4.4 | 86.4±2.9 | 84.8±4.6 | 17.5±4.2 | 33.3±0 | 16.3±2.1 | 33.3±0 | -33.9±3.8 |
| P7304 | Exponential | 2.4±3.5 | 1.6±0.6 | 12.2±17.1 | 72.6±6 | 75±0 | 72±1 | 52.9±0 | -26.4±4.2 |
| SK11 | Exponential | 93.9±1.6 | 92.2±0.5 | 92.3±1.5 | 6.3±0.7 | 51.8±16.2 | 7.8±0.7 | 51.8±16.2 | -19.3±0.3 |
| SK110 | Exponential | 94.5±2.8 | 92.9±2.6 | 94.5±2.6 | 37±1.5 | 58.3±2.8 | 41±0.7 | 58.3±2.8 | -30.5±1.8 |
| TIFN1 | Exponential | 95.9±1.1 | 96.8±0.5 | 92.8±0.5 | 24.3±3.5 | 0±0 | 27.3±1.9 | 0±0 | -7.2±1.3 |
| TIFN2 | Exponential | 91.7±4.4 | 93.1±4 | 92.5±2.8 | 11.7±8.1 | 0±0 | 37±0.9 | 0±0 | -13.5±1.1 |
| TIFN3 | Exponential | 93±3 | 91.6±3.3 | 88.2±4.2 | 80.1±6.8 | 68.8±0 | 80.8±5.9 | 68.8±0 | -13.4±1 |
| TIFN4 | Exponential | 63±13.5 | 79.3±8.4 | 90±3.4 | 15.1±7.2 | 0±0 | 7.2±1 | 0±0 | -13.5±0.8 |
| TIFN5 | Exponential | 91.1±3.2 | 96.8±0.9 | 95.8±1.1 | 10.8±1.9 | 58.1±50.3 | 12.8±2.1 | 58.1±50.3 | -7±0.1 |
| TIFN6 | Exponential | 94.3±2.1 | 95.8±0.7 | 94.4±0.5 | 25.6±3.7 | 0±0 | 24.4±2.7 | 0±0 | -7.2±0.1 |
| TIFN7 | Exponential | 93.6±3.6 | 79.4±1.6 | 95.5±2.1 | 21.2±1.1 | 15.9±3.6 | 21.9±2.8 | 15.9±3.6 | -13.9±0.8 |
| UC317 | Exponential | 96.5±0.3 | 96.4±0.6 | 96.4±0.2 | 63.2±7.3 | 17.4±0 | 77.5±3.3 | 22.2±0 | -3.3±0.1 |
| V4 | Exponential | 2.4±4 | 0.4±0.4 | 1.6±0.5 | 82±2 | 87.5±0 | 56.7±1.2 | 87.5±0 | -13±2.8 |
| AM2 | Stationary | 94.9±1.2 | 94.5±0.4 | 94.9±1.7 | 3.7±1.2 | 76.9±40 | 2.2±0.8 | 76.9±40 | -25±1.6 |
| ATCC19435 | Stationary | 84.6±4.8 | 87.6±2.1 | 90.2±1.5 | 7.3±1.1 | 38.9±0 | 4.4±0.5 | 35±0 | -21.3±1.2 |
| B40 | Stationary | 98.4±1.1 | 99.3±0.3 | 99.2±0.1 | 52.3±11.1 | 60.3±3.4 | 44.6±7.2 | 60.3±3.4 | -26.6±1.8 |
| DRA4 | Stationary | 89.6±2.2 | 91.7±1.4 | 89.4±2 | 0.5±0.2 | 0±0 | 0.3±0.1 | 0±0 | -33.4±3.2 |
| E34 | Stationary | 15.4±5.9 | 17.5±5.7 | 14.8±9.5 | 93.7±1.7 | 46.7±0 | 93.7±1 | 53.3±0 | -21.3±1.6 |
| FG2 | Stationary | 9.5±3.6 | 9.1±3.4 | 91.8±1.4 | 79.5±5.6 | 81.3±0 | 62.5±0.9 | 68.8±0 | -7±1.8 |
| HP | Stationary | 95.5±1.9 | 95.9±1.4 | 95±1.2 | 12.6±2 | 58.3±0 | 3±0.6 | 58.3±0 | -20.9±0.5 |
| IL1403 | Stationary | 63.7±1.6 | 41.6±13.6 | 67.2±2.2 | 0.7±0.9 | 66.3±8.9 | 4.3±1.2 | 66.3±8.9 | -7.1±0.1 |
| K231 | Stationary | 9.2±2.4 | 8.3±3.7 | 11.2±5 | 2.9±0.1 | 61.7±12.6 | 2.8±0.2 | 61.7±12.6 | -39.4±1.6 |
| K337 | Stationary | 0±0 | 45.4±8.3 | 45.2±16.8 | 97.3±0.4 | 66.7±0 | 93.9±1.9 | 75±0 | -23.4±3.3 |
| KF134 | Stationary | 51.4±4 | 52.6±3.2 | 57.5±2.4 | 42.1±4 | 33.3±0 | 44.1±3 | 43.8±0 | -4.1±0.4 |
| KF146 | Stationary | 86.7±1.7 | 82.5±2.8 | 87±1.4 | 76.2±0.3 | 76.9±0 | 79.5±5.2 | 87.5±0 | -7.1±0.5 |
| KF147 | Stationary | 50.3±1.2 | 61.2±2 | 46.4±10.4 | 13±3 | 0±0 | 10.6±1 | 0±0 | -3.8±0.6 |
| KF196 | Stationary | 71.2±9.9 | 68.3±1 | 69.1±2.1 | 58.4±3.9 | 33.3±0 | 62.5±1.2 | 53.3±0 | -4.8±0.6 |
| KF201 | Stationary | 8.1±1.5 | 15.1±7.7 | 5.1±5.5 | 94±4.3 | 100±0 | 94.2±0.5 | 100±0 | -25±0.5 |
| KF24 | Stationary | 79.6±5 | 79.7±2.7 | 79.9±4.1 | 14.9±20.3 | 70.6±0 | 36.7±0.5 | 88.9±0 | -28.3±0.4 |
| KF282 | Stationary | 60.7±0.7 | 63.9±1.7 | 64.5±0.9 | 95±1.5 | 82.4±0 | 94.3±0.6 | 82.4±0 | -25±0.9 |
| KF67 | Stationary | 55.8±1.5 | 61.2±1.7 | 60.3±2.9 | 22.1±1.6 | 73.3±0 | 22.9±1.3 | 73.3±0 | -36.1±0.8 |
| KF7 | Stationary | 12.1±11.7 | 13.8±3.8 | 1.9±3.2 | 2.8±0.4 | 8.3±0 | 2±0.8 | 8.3±0 | -36.9±1.3 |
| KW10 | Stationary | 10.9±6.9 | 11.3±2.1 | 9.2±4.2 | 93.5±0.8 | 93.8±0 | 96.9±0.7 | 83.3±0 | -34±2.6 |
| Li-1 | Stationary | 14.8±2.4 | 18.4±1.7 | 17.9±6.6 | 34±7.5 | 20±0 | 34.4±3.5 | 42.9±0 | -35.4±0.9 |
| LMG14418 | Stationary | 31±20.7 | 13.6±3 | 23.2±24 | 16.3±6.5 | 0±0 | 11.2±1.9 | 0±0 | -38.3±1.9 |
| LMG6897 | Stationary | 78.1±0.5 | 79.7±1.2 | 79.2±0.8 | 87±3.7 | 68.8±0 | 89.8±1.8 | 68.8±0 | -28.2±0.7 |
| LMG8520 | Stationary | 70.9±9.2 | 81.3±2.5 | 83.8±3.4 | 47.3±9.2 | 16.7±0 | 37.8±17.9 | 41.4±0 | -24.8±4 |
| LMG8526 | Stationary | 88.4±0.8 | 91.9±0.1 | 91.9±0.9 | 3.7±0.2 | 42.8±14.4 | 3.2±0.2 | 42.8±14.4 | -30.2±0.3 |
| LMG9446 | Stationary | 89.8±1 | 90.3±2.2 | 90.9±4.5 | 13.5±0.4 | 0±0 | 13.1±0.3 | 0±0 | -24±0.7 |
| LMG9447 | Stationary | 86.1±3.9 | 82.8±4.4 | 84.9±7.7 | 1±0.3 | 0±0 | 0.9±0.3 | 0±0 | -42.5±6.6 |
| M20 | Stationary | 86.4±1.7 | 87.6±3.3 | 88.6±3 | 86.8±7.7 | 57.1±0 | 94.5±1.4 | 35.7±0 | -20.1±0.6 |
| MG1063 | Stationary | 83±5.1 | 89.5±2.6 | 89.6±2.1 | 16.7±0 | 81.3±0 | 10.3±0.5 | 62.5±0 | -29±0.9 |
| MG1261 | Stationary | 88.8±6.3 | 94±1.4 | 92.5±0.8 | 21.8±0.2 | 52.9±0 | 8.9±1.5 | 46.7±0 | -30±1.2 |
| MG1299 | Stationary | 79.2±2.2 | 82.8±2.7 | 82.7±0.3 | 65.2±6.5 | 53.3±0 | 79±2.4 | 40±0 | -38.3±2.1 |
| MG1362 | Stationary | 79.6±14.1 | 89.6±3.4 | 84.9±9.7 | 5.6±9.6 | 68.8±0 | 9.2±1.6 | 50±0 | -32.6±2 |
| MG1363 | Stationary | 79.4±1.7 | 82.3±0.9 | 82.8±2 | 5.8±0.2 | 0±0 | 8.1±0.2 | 0±0 | -30.2±0.7 |
| MG1365 | Stationary | 76.6±26.4 | 92.9±1.8 | 93.1±1.3 | 28.6±6.1 | 62.5±0 | 12.7±1.3 | 43.8±0 | -30.3±1.2 |
| ML8 | Stationary | 17.1±6.4 | 19.8±5.6 | 13.9±4.6 | 26.3±12.3 | 88.2±0 | 42.1±9.2 | 88.2±0 | -11.3±0.8 |
| N41 | Stationary | 15.3±10.1 | 18.1±1.2 | 17.5±15.2 | 69.5±9 | 28.6±0 | 87.5±5.4 | 42.9±0 | -34.3±0.3 |
| N42 | Stationary | 90.2±1.9 | 85.8±3.6 | 91.8±0.2 | 96.8±1.6 | 73.3±0 | 100±0 | 33.3±0 | -19.4±1 |
| NCDO712 | Stationary | 94.7±0.6 | 96±0.6 | 95.7±2 | 99.4±0.3 | 100±0 | 99±0.6 | 100±0 | -21.1±1.9 |
| NCDO763 | Stationary | 92.5±1.4 | 92±1.8 | 92.6±0.5 | 28±1.2 | 0±0 | 32.5±4.1 | 0±0 | -30.2±0.6 |
| NCDO895 | Stationary | 81.2±4.1 | 86.1±5.2 | 85.2±1.5 | 75.6±12.5 | 85±0 | 94.2±0.3 | 71.4±0 | -12.9±0.2 |
| NIZO2244B | Stationary | 41.6±0.8 | 26.5±15.6 | 32.2±18.1 | 7.9±4.1 | 0±0 | 1.2±0.5 | 0±0 | -39.4±1.3 |
| NZ9000 | Stationary | 65.8±23.6 | 65.8±6.9 | 65.2±6.9 | 13.3±2 | 36.5±33.8 | 13±2.2 | 36.5±33.8 | -33.1±1.5 |
| P7266 | Stationary | 0.2±0.4 | 2.2±3.6 | 0±0 | 44.5±3.2 | 33.3±0 | 37.5±3.4 | 33.3±0 | -36.8±2 |
| P7304 | Stationary | 16.6±4.4 | 18.6±6.3 | 33.9±29.1 | 94.7±0.8 | 68.8±0 | 94.8±1 | 37.5±0 | -23±1.2 |
| SK11 | Stationary | 96.6±0.1 | 97.4±0.3 | 95.4±1.7 | 7.8±1.7 | 0±0 | 3±1.5 | 0±0 | -18.9±1.2 |
| SK110 | Stationary | 93±6 | 97.6±0.6 | 97.2±1.5 | 7.5±0.3 | 63.3±20.2 | 28.8±1.3 | 63.3±20.2 | -23.3±0.9 |
| TIFN1 | Stationary | 96.6±0.4 | 97.5±0.1 | 97.7±0.3 | 20.5±0.2 | 0±0 | 7.9±0.9 | 0±0 | -6.5±0.1 |
| TIFN2 | Stationary | 84.2±10.3 | 91.5±0.6 | 93.4±2.4 | 5.3±3.3 | 0±0 | 4.2±1.6 | 0±0 | -15±1.3 |
| TIFN3 | Stationary | 82.5±22.5 | 93.4±1.9 | 94.2±2.1 | 68.5±3.8 | 68.8±0 | 70.7±6.3 | 68.8±0 | -8.2±0.5 |
| TIFN4 | Stationary | 78.6±14.5 | 84.3±8.1 | 79.3±3.4 | 1.5±0.6 | 0±0 | 1.9±0.5 | 0±0 | -15.9±0.4 |
| TIFN5 | Stationary | 98.6±0.7 | 98.3±0.3 | 98.1±0.8 | 4±1.6 | 0±0 | 2.7±0.3 | 0±0 | -13.4±1.9 |
| TIFN6 | Stationary | 80.4±25.1 | 97±0.8 | 96.8±0.7 | 3.1±0.6 | 0±0 | 3±0.5 | 0±0 | -8.8±0.3 |
| TIFN7 | Stationary | 92.6±1.6 | 89.8±0.1 | 92.9±4.3 | 2.6±0.3 | 0±0 | 2.3±0.2 | 0±0 | -15.3±1.6 |
| UC317 | Stationary | 88.5±0.4 | 92.6±0.5 | 91±3.4 | 76.6±2.5 | 11.8±0 | 63±6.2 | 36±0 | -3.4±1.1 |
| V4 | Stationary | 22.7±2 | 12.3±2.7 | 14.2±4.6 | 88.8±3.1 | 87.5±0 | 69.2±4.6 | 87.5±0 | -20.4±1.3 |

**Table S 3. The 18 genes selected for further characterization.**

| **Protein name /locus tag of gene** | **Locus_tag** | **Modification** | **Growth phase ^N^** | **Gene**  **Presence (Pr) or Absence (Ab)** | **Predicted phenotype change^A^** |
| --- | --- | --- | --- | --- | --- |
| Cell surface protein precursor/  *B40_0084* | B40: locus_tag=B40_0084 | Overexpression in MG1363 from B40 (930 aa) | ST | Pr | ZP ▼ |
| Cell wall surface anchor family protein/  *pLP712_21* | MG1299: locus_tag=pLP712_21 | Overexpression in MG1363 from pLP712 of MG1299 (371 aa) | EX | NA | CSH ▲ |
| Ribose 5-phosphate isomerase A/  *KF147_0667* | KF147 locus_tag=KF147RAST_0688 | Overexpression in MG1363 from KF147 (234 aa) | EX | Ab | ParaCN ▼ |
| Possible surface protein/  *LLKF_0684* | Locus tag in KF147: LLKF_0684 | Overexpression in MG1363 from from KF147 (999aa) | ST | Pr | ZP ▼ |
| Cell wall surface anchor family protein/  *llmg_1148* | MG1363: locus_tag=llmg_1148; NZ9000: locus_tag=LLNZ_05925 | Knockout in MG1363 (567 aa) | ST | Ab | ZP ▼ |
| Iinternalin, putative (LPXTG motif)/ *IL1403RAST_1416* | IL1403: locus_tag=IL1403RAST_1416 | Overexpression in MG1363 from IL1403 (653 aa) | EX | Pr | CSH ▲ |
| Internalin, putative (LPXTG motif)/  *KF282_0409* | KF282: locus_tag=0409 | Overexpression in MG1363 from KF282 (559 aa) | ST | Ab | ZP ▼ |
| Cell wall surface anchor family protein/  *llmg_0009* | MG1363RAST_0009 locus_tag=llmg_0009 | Knockout in MG1363 (227 aa) | EX | Ab | ParaCN ▼  CSH ▲ |
|  |  |  |  | Ab |  |
| Hypothetical protein/  *L128699* | Locus tag: IL1403RAST_1821 | Overexpression in MG1363 from IL1403 (yreB, 314 aa) | EX | Pr | CSH ▲ |
| Hypothetical protein/  *D688_p3025* | MG1363: locus_tag=D688_p3025 | Knockout in MG1363 (TraD, 612 aa) | EX | Ab | ParaCN ▼ |
| Cell wall surface anchor family protein/  *LLKF_0311* | KF147: locus_tag=LLKF_0311 | Overexpression in MG1363 from KF147 (809 aa) | EX | Pr | CSH ▲  E24 ▲ |
|  |  |  |  | Ab |  |
| Xyloside transporter/*XynT* | IL1403 : locus_tag=L0233 | Overexpresion in MG1363 from IL1403 (490 aa) | ST | Ab | ParaCN ▲  NaCN ▲ |
|  |  |  |  | Ab |  |
| Cell surface protein precursor/  *llmg_1096* | MG1363: locus_tag=llmg_1096; NZ9000: LLNZ_05670; MG1299~llmg_1096 | Knockout in MG1363 (387 aa) | ST | Ab | ZP ▼ |
| Extracellular protein/ *llmg_1095* | IL1403: locus_tag=L191998; NZ9000~LLNZ_05665; MG1299~llmg_1095; MG1363: locus_tag=llmg_1095 | Knockout in MG1363 (247 aa) | ST | Ab | ZP ▼ |
| endo-beta-N-acetylglucosaminidase /  *LLKF_1605* | KF147 locus_tag=LLKF_1605 | Overexpression in MG1363 from KF147 (923 aa), gene="ypcCD" | ST | Ab | ParaCN ▲  NaCN ▲ |
|  |  |  |  | Ab |  |
| Hypothetical protein/  *llmg_1093* | IL1403: locus_tag=193176; MG1363: locus_tag=llmg_1093; NZ9000~LLNZ_05655; MG1299~llmg_1093 | Knock-out in MG1363 (334 aa) | ST | Pr | NaCN ▲ |
| Sortase A*/srtA/* *llmg_1449* | locus_tag=llmg_1449 | Knock-out in MG1363 (250 aa) |  | Manual selection | NA |
| Sortase C*/ srtC/* *llmg_1801* | locus_tag=llmg_1801 | Knockout in MG1363 |  | Manual selection | NA |

^N^ Data for genotype-phenotype matching was obtained from cells that originated from the indicated growth phase; EX-Exponential growth phase, ST –stationary growth phase

^A^ ZP – charge (mV), ParaCN – attachment to para-caseinate (%), NaCN – attachment to sodium caseinate (%), NaCN90C – attachment to sodium caseinate heated at 90C for 10 min (%), CSH – cell surface hydrophobicity (%), E24 – emulsion stability for 24h (%), NA – not applicable, ▲ – cell surface property increases, ▼ – cell surface property decreases.

**Table S 4. Cell surface properties of the 10 obtained mutants.** The measured properties are cell surface hydrophobicity (CSH, %) measured with petroleum, emulsion stability after 24 h (E24, %), charge (ZP, mV), attachment to sodium caseinate (NaCN, %), to the same protein heated for 10 min at 90°C (NaCN90C, %), and to para-caseinate (ParaCN, %). The results represent the average of 6 measurements and standard deviation. Significance was calculated using a two-tailed distribution t-test comparing surface properties of each strain to its control in the corresponding growth phase; * p<0.01

| **Strain** | **Protein** | **ParaCN, %** | **NaCN, %** | **NaCN90C, %** | **CSH, %** | **E24, %** | **ZP, mV** |
| --- | --- | --- | --- | --- | --- | --- | --- |
| Overexpression in *L. lactis* NZ9000pNZ8150 | | | | | | | |
| Exponential growth phase | | | | | | | |
| NZ9000pNZ8150 |  | 98.9±0.5 | 99.2±0.5 | 98.9±0.4 | 11.1±13.9 | 0±0 | -29.3±2.4 |
| NZ9000pNZ8150_*B40_0084* | Cell surface protein precursor | 99.3±0.6 | 98.9±1.1 | 99.3±0.3 | 23.7±26.0 | 0±0 | -25.3±1.5 |
| NZ9000pNZ8150_*KF147_0667* | Ribose 5-phosphate isomerase A | 98.1±1.5 | 98.9±0.3 | 98.7±0.5 | 4.1±1.8 | 0±0 | -27.5±1.1 |
| NZ9000pNZ8150*_KF282_0409* | Internalin_putative_LPXTG motif | 96.3±4.6 | 94.2±11.6 | 95.5±5.6 | 49.6±6.3 | 8.3±20.4 | -24.7±1.9 |
| NZ9000pNZ8150*_L128699* | Hypothetical protein | 98.9±0.8 | 99.2±0.4 | 98.3±2.5 | 2.6±0.9 | 0±0 | -26.6±2.5 |
| NZ9000pNZ8150_*LLKF_0311* | Cell wall surface anchor family protein | 32.4±50.3^*^ | 43.1±47.6^*^ | 38.6±48.3^*^ | 40.7±35.9 | 0±0 | -25.2±1.7 |
| NZ9000pNZ8150_*LLKF_1605* | Endo-beta-N-acetylglucosaminidase | 98.5±1.0 | 98.8±0.5 | 99.2±0.6 | 2.4±2.3 | 0±0 | -26.5±1.0 |
| Stationary growth phase | | | | | | | |
| NZ9000pNZ8150 |  | 97.9±0.1 | 98.7±0.6 | 97.9±0.7 | 8.8±5.0 | 0±0 | -27.4±1.5 |
| NZ9000pNZ8150*_B40_0084* | Cell surface protein precursor | 98.9±0.8^*^ | 99.8±0.1^*^ | 99.7±0.2^*^ | 89.4±6.8^*^ | 58.6±25.9 | -25.1±1.3 |
| NZ9000pNZ8150*_KF147_0667* | Ribose 5-phosphate isomerase A | 99.1±0.5^*^ | 99.5±0.1^*^ | 99.5±0.2^*^ | 4.8±2.6 | 0±0 | -27.8±3.6^*^ |
| NZ9000pNZ8150_*KF282_0409* | Internalin_putative_LPXTG motif | 99.1±0.3^*^ | 94.5±11.9^*^ | 99.3±0.7^*^ | 49.3±21.8 | 31.4±35.1 | -27.1±1.6 |
| NZ9000pNZ8150_*L128699* | Hypothetical protein | 98.9±1.0^*^ | 98.7±1.1^*^ | 98.3±1.8^*^ | 2.4±4.5 | 0±0 | -28.2±0.7 |
| NZ9000pNZ8150_*LLKF_0311* | Cell wall surface anchor family protein | 31.2±48.4^*^ | 29.9±46.4^*^ | 31.5±48.8^*^ | 78.3±9.0^*^ | 0±0 | -26.8±0.7 |
| NZ9000pNZ8150*_LLKF_1605* | Endo-beta-N-acetylglucosaminidase | 99.5±0.3^*^ | 99.5±0.3^*^ | 99.6±0.1^*^ | 23.7±16.1 | 0±0 | -27.3±0.9 |
| Knockout in *L. lactis* MG1363 | | | | | | | |
| Exponential growth phase | | | | | | | |
| MG1363 |  | 96.6±1.7 | 98.0±0.3 | 95.6±2.4 | 6.3±3.1 | 0±0 | -25.6±0.9 |
| MG1363∆*llmg_1383* | **C**onjugal transfer protein **(TraG)** | 41.9±6.4 | 48.3±9.6 | 42.8±4.7 | 7.7±2.5 | 0±0 | -29.1±0.7 |
| MG1363∆*llmg_1096* | Cell surface protein precursor | 98.6±0.4 | 98.9±0.5 | 98.9±0.3 | 6.9±4.2 | 0±0 | -27.8±0.4 |
| MG1363∆*llmg_1093* | Hypothetical protein | 25.5±22.3 | 55.9±35.0 | 28.9±25.2 | 1.2±2 | 0±0 | -28.8±0.5 |
| MG1363∆*llmg_1449* | Sortase A | 98.3±0.6 | 98.9±0.2 | 97.5±1.6 | 7.9±2.8 | 0±0 | -29.9±1.1 |
| Stationary growth phase | | | | | | | |
| MG1363 |  | 79.4±1.7 | 82.3±0.9 | 82.8±1.9 | 5.8±0.2 | 0±0 | -30.2±0.7 |
| MG1363∆*llmg_1383* | **C**onjugal transfer protein **(TraG)** | 95.9±3.9 | 85.5±11.9 | 97.7±0.7 | 0.9±1.6 | 0±0 | -28.8±2.5 |
| MG1363∆*llmg_1096* | Cell surface protein precursor | 98.8±0.4 | 99.4±0.1 | 99.2±0.4 | 5.3±4.9 | 0±0 | -29.3±0.9 |
| MG1363∆*llmg_1093* | Hypothetical protein | 47.5±48.9 | 25.4±22.4 | 43.6±39.7 | 6.9±6.7 | 0±0 | -29.5±1.8 |
| MG1363∆*llmg_1449* | Sortase A | 98.8±0.5 | 99.1±0.4 | 99.2±0.1 | 0.5±1.3 | 0±0 | -28.9±0.9 |
